# Derepression of inflammation-related genes link to microglia activation and neural maturation defect in a mouse model of Kleefstra syndrome

**DOI:** 10.1016/j.isci.2021.102741

**Published:** 2021-06-17

**Authors:** Ayumi Yamada, Takae Hirasawa, Kayako Nishimura, Chikako Shimura, Naomi Kogo, Kei Fukuda, Madoka Kato, Masaki Yokomori, Tetsutaro Hayashi, Mana Umeda, Mika Yoshimura, Yoichiro Iwakura, Itoshi Nikaido, Shigeyoshi Itohara, Yoichi Shinkai

**Affiliations:** 1 Cellular Memory Laboratory, Cluster for Pioneering Research, RIKEN, Wako, Saitama, Japan; 2 Department of Biosciences, School of Science and Engineering, Teikyo University, 1-1 Toyosatodai, Utsunomiya, Tochigi, Japan; 3 Laboratory for Behavioral Genetics, RIKEN Center for Brain Science, Wako, Saitama, Japan; 4 Laboratory for Bioinformatics Research, RIKEN Center for Biosystems Dynamics Research, Wako, Saitama, Japan; 5 Research Institute for Biomedical Sciences, Tokyo University of Science, Noda, Chiba 278-8510, Japan; 6 Functional Genome Informatics, Medical Research Institute, Tokyo Medical and Dental University, Bunkyo, Tokyo, Japan; 7 Bioinformatics Course, Master's/Doctoral Program in Life Science Innovation (T-LSI), School of Integrative and Global Majors (SIGMA), University of Tsukuba, Wako, Saitama 351-0198, Japan

**Keywords:** Pathophysiology, Behavioral neuroscience, Molecular neuroscience, Developmental neuroscience

## Abstract

Haploinsufficiency of *EHMT1*, which encodes histone H3 lysine 9 (H3K9) methyltransferase G9a-like protein (GLP), causes Kleefstra syndrome (KS), a complex disorder of developmental delay and intellectual disability. Here, we examined whether postnatal supply of GLP can reverse the neurological phenotypes seen in *Ehmt1*^Δ/+^ mice as a KS model. Ubiquitous GLP supply from the juvenile stage ameliorated behavioral abnormalities in *Ehmt1*^Δ/+^ mice. Postnatal neuron-specific GLP supply was not sufficient for the improvement of abnormal behaviors but still reversed the reduction of H3K9me2 and spine number in *Ehmt1*^Δ/+^ mice. Interestingly, some inflammatory genes, including *IL-1β (Il1b)*, were upregulated and activated microglial cells increased in the *Ehmt1*^Δ/+^ brain, and such phenotypes were also reversed by neuron-specific postnatal GLP supply. *Il1b* inactivation canceled the microglial and spine number phenotypes in the *Ehmt1*^Δ/+^ mice. Thus, H3K9me2 and some neurological phenotypes are reversible, but behavioral abnormalities are more difficult to improve depending on the timing of GLP supply.

## Highlights

• Activated microglias increase in a *Ehmt1*^Δ/+^ mouse model of Kleefstra syndrome
• Diminished H3K9me2 in *Ehmt1*^Δ/+^ mouse neurons is reversed by post-natal GLP supply
• GLP supply from the juvenile stage can improve abnormal behaviors of *Ehmt1*^Δ/+^ mice
• *Il1b* KO cancelles the microglial and spine number phenotypes in the *Ehmt1*^Δ/+^ mice

## Graphical abstract

**Figure:**
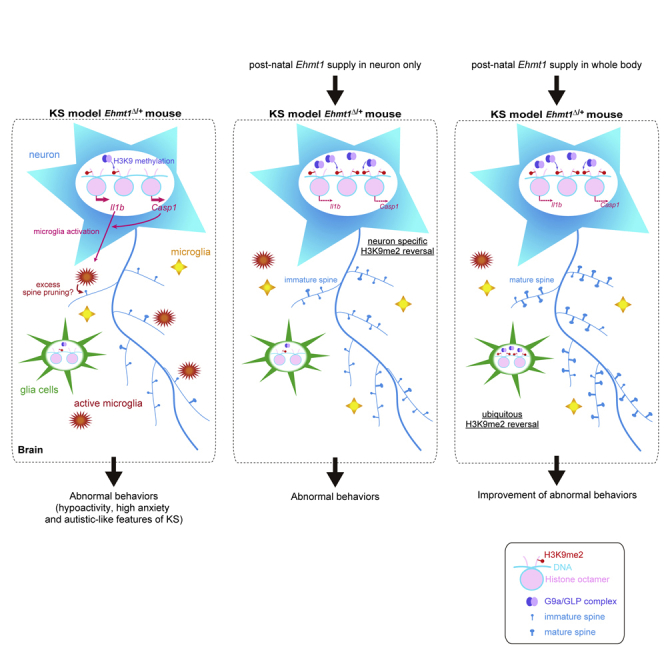

## Introduction

Kleefstra syndrome (KS), also known as 9q.34.3 deletion syndrome, is a rare genetic disorder characterized by developmental delay, intellectual disability, and autistic-like features (Kleefstra and de Leeuw, 2010). KS is caused by haploinsufficiency of the gene named euchromatic histone lysine methyltransferase 1 (*EHMT1*) (Iwakoshi et al., 2004; Kleefstra et al., 2006) which encodes a lysine methyltransferase G9a-like protein (GLP) (also known as KMT1D). GLP forms a complex with another lysine methyltransferase G9a (also known as EHMT2 or KMT1C), and this heteromeric complex regulates histone H3 lysine 9 dimethylation (H3K9me2), which is a well-conserved epigenetic mark (epigenome) (Shinkai and Tachibana, 2011). Our knowledge of how *EHMT1* haploinsufficiency causes KS is still very limited; thus, designing a causal treatment protocol remains challenging. KS is a complex neurodevelopmental disorder comprising many neuropsychiatric and somatic phenotypes that differ as one ages along the lifespan (Stewart and Kleefstra, 2007; Willemsen et al., 2012). Some of the phenotypes can be treated for improvement or managed, but development of more fundamental treatment methods is still essential. To design such a treatment strategy, it is critical to know how much *EHMT1* haploinsufficiency-mediated KS phenotypes can be reversed or suppressed by postnatal supply of *EHMT1* or the correction of epigenome dysregulation. If insufficient GLP lysine methyltransferase activity is essential for the majority of KS phenotypes, manipulation of epigenome regulation is a reasonable direction for future KS treatment. *Ehmt1* heterozygous knockout (KO) (*Ehmt1*^Δ/+^) or neuron-specific *Ehmt1* KO mice show hypoactivity and autistic-like features of KS, although brain development and architecture are grossly normal (Balemans et al., 2010; Schaefer et al., 2009). Although a previous study in a *Drosophila* model of KS (a fly mutant of *EHMT*; an ortholog of mammalian *EHMT1* or *EHMT2*) showed that defects in cognitive capabilities (memory) can be restored by *EHMT* re-expression during adulthood (Kramer et al., 2011), similar studies in mammalian models of KS have not been reported. In this study of *Ehmt1*^Δ/+^ mice as a KS model, we investigated whether neurological phenotypes, including abnormal behaviors of *Ehmt1*^Δ/+^ mice, can be reversed by postnatal GLP supply. In addition, we showed that neural inflammation may play a role in the molecular pathology of *Ehmt1*^Δ/+^ mouse brains.

## Results

### Postnatal GLP supply to the entire brain from the juvenile stage can improve abnormal behaviors of *Ehmt1*^Δ/+^ mice

**Figure 1:**
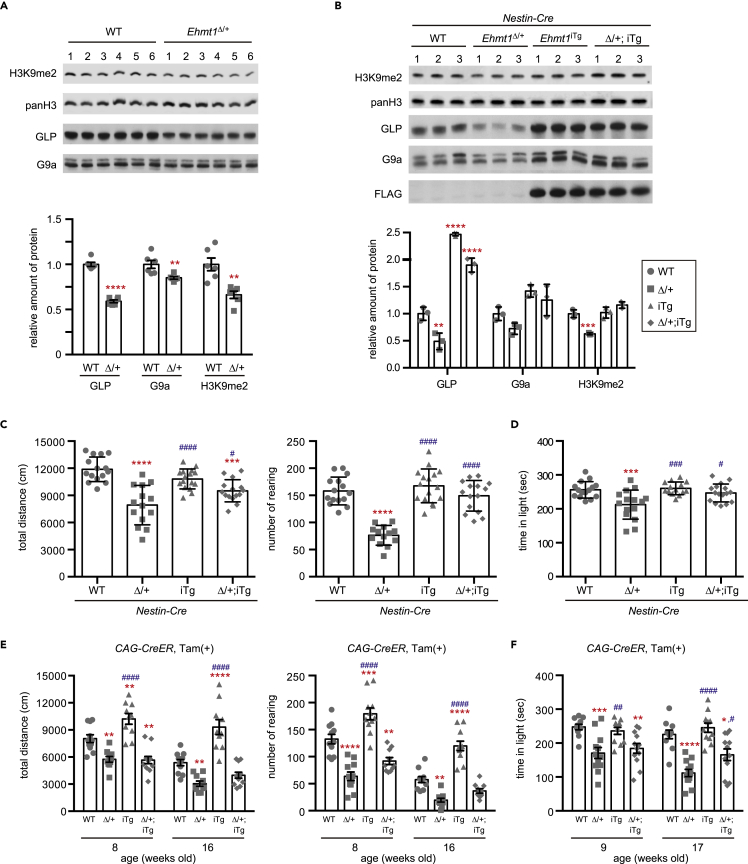
GLP supply to brain from a prenatal stage or postnatal whole body can improve abnormal behaviors in *Ehmt1*^Δ/+^ mice (A) Western blot analysis of GLP, G9a, and Histone H3K9me2 in wild-type (WT, circle) and *Ehmt1*^Δ/+^ (Δ/+, square) mouse brain lysate (8 weeks old). H3K9me2 level and amount of G9a and GLP were normalized with pan Histone H3. Normalized individual data was calculated as the mean of WT as 1 and plotted with mean ± SD (n = 6). ^∗∗^p < 0.01, ^∗∗∗∗^p < 0.0001 (vs WT), unpaired t test. (B) Western blot analysis of GLP, G9a, FLAG (FLAG-GLP), Histone H3K9me2, and pan Histone H3 in brain lysate of wild-type (WT, circle), *Ehmt1*^Δ/+^ (Δ/+, square), *Ehmt1* iTg (iTg, up triangle) and *Ehmt1*^Δ/+^; iTg (Δ/+; iTg, diamond) with *Nestin-Cre* (16 weeks old). Protein amount of GLP, G9a, and H3K9me2 level was normalized with pan Histone H3. Normalized individual data calculated as the mean of WT as 1 was plotted with mean ± SD (n = 3). ^∗∗^p < 0.01, ^∗∗∗^p < 0.001 and ^∗∗∗∗^p < 0.0001 (vs WT), One-way ANOVA. (C) Open field test of wild-type (WT, circle), *Ehmt1*^Δ/+^ (Δ/+, square), *Ehmt1* iTg (iTg, up triangle), and *Ehmt1*^Δ/+^; iTg (Δ/+; iTg, diamond) with *Nestin-Cre* mice was performed at 6 weeks old. Total distance (cm) (left) and the rearing number (right) were shown as mean ± SD (n = 14–16). ^∗∗∗^p < 0.001, ^∗∗∗∗^<0.0001 (vs WT) and ^#^p < 0.05, ^####^p < 0.0001 (iTg or Δ/+; iTg vs *Ehmt1*^Δ/+^), One-way ANOVA. (D) Light/dark test of *Nestin-Cre* mouse group (7 weeks old). Staying time in light (sec) was shown as mean ± SD. ^∗∗∗^p < 0.001 (vs WT) and ^#^p < 0.05, ^###^p < 0.001 (iTg or Δ/+; iTg vs *Ehmt1*^Δ/+^), One-way ANOVA. (E) Open field test of wild-type (WT, circle), *Ehmt1*^Δ/+^ (Δ/+, square), *Ehmt1* iTg (iTg, up triangle) and *Ehmt1*^Δ/+^; iTg (Δ/+; iTg, diamond) with *CAG-CreER* mice treated with Tam at 3 and 4 weeks old (n = 9–12) was examined at 8 or 16 weeks old. Total distance (cm) (left) and the rearing number (right) were shown as mean ± SD (n = 7–9). ^∗∗^p < 0.01, ^∗∗∗^p < 0.001 and ^∗∗∗∗^p < 0.0001 (vs WT), ^####^p < 0.0001 (iTg or Δ/+; iTg vs Δ/+), repeated two-way ANOVA. (F) Light/dark test of *CAG-CreER* mouse group was analyzed at 9 weeks old or 17 weeks old. Staying time in light (sec) was shown as mean ± SD. ^∗^p < 0.05, ^∗∗^p < 0.01, ^∗∗∗^p < 0.001, ^∗∗∗∗^p < 0.0001 (vs WT) and ^#^p < 0.05, ^##^p < 0.01 and ^####^p < 0.0001 (iTg or Δ/+; iTg vs Δ/+), repeated two-way ANOVA. See also Figures S1, S2, and S6.

Before we examined whether postnatal GLP supply can rescue KS phenotypes, we first confirmed that *Ehmt1* haploinsufficiency impacts H3K9me2 in the brain at a global level. It has been reported that H3K9me2 immunostaining signals decreased in *Ehmt1*^Δ/+^ brain sections (Benevento et al., 2016) and that the levels of H3K9me2 and GLP/EHMT1 expression diminished in *in vitro* induced neurons derived from induced pluripotent stem (iPS) cells originating from patients with KS (Frega et al., 2019). As shown in Figure 1A, the amount of GLP in the *Ehmt1*^Δ/+^ brain decreased to ∼60% of that in the wild-type (WT). The amount of G9a also diminished to ∼80% of the WT level, which is consistent with the finding that GLP stabilizes G9a protein (Tachibana et al., 2005). The global level of H3K9me2 in the *Ehmt1*^Δ/+^ brain also diminished to ∼70% of that in the WT brain (Figure 1A).

We first examined whether exogenous GLP supply from the embryonic stage could rescue the diminished H3K9me2 and abnormal behavioral phenotypes of *Ehmt1*^Δ/+^ mice. To address this issue, we utilized the conditional FLAG-tagged GLP-inducible line, *Rosa26-CAG-FLAG-Ehmt1* transgenic (Tg) (*Ehmt1 iTg*), in which FLAG-GLP expression was induced once Cre recombinase was expressed in the nucleus (Figure S1A) (Deguchi et al., 2013). Using the different Cre or inducible Cre Tg lines, GLP expression can be controlled in a tissue-specific and time-dependent manner. We first used *Nestin-Cre* Tg mice as a Cre driver, in which Cre is expressed in neural stem cells from the prenatal stage (Isaka et al., 1999). FLAG-GLP was detected in both neural and non-neural cells in *Ehmt1 iTg* mice brains (Figure S1B). We analyzed H3K9me2 levels in brain lysates from these lines, and reduced H3K9me2 in *Ehmt1*^Δ/+^ mice was recovered by GLP re-expression in *Ehmt1*^Δ/+^; *iTg* mice (Figure 1B). We performed behavioral tests with *Nestin-Cre* mice (Figures 1C and 1D). It has been reported that *Ehmt1*^Δ/+^ mice show abnormal behaviors, such as reduced locomotor activity and elevated anxiety (Balemans et al., 2010; Schaefer et al., 2009), and we also confirmed the same phenotypes in *Ehmt1*^Δ/+^ mice. Behaviors of *Ehmt1*^Δ/+^; *iTg* with *Nestin-Cre* mice were clearly improved or rescued at the wild-type level. The results demonstrated that the diminished H3K9me2 and KS-like abnormal behavioral phenotypes of the *Ehmt1*^Δ/+^ mice could be reversed if the GLP was supplied to the brain from the prenatal stage and also indicated that the abnormal behavioral phenotypes were mostly caused by haploinsufficiency of *Ehmt1* in the brain.

Since we learned that sufficient supply of GLP into neural cells from the embryonic stage could rescue the behavioral phenotypes of the *Ehmt1*^Δ/+^ mice, we next addressed how much the abnormal behavioral and hypo H3K9me2 phenotypes of the *Ehmt1*^Δ/+^ mice could be rescued by postnatal GLP supply. To address this problem, we first used *CAG-CreER* Tg mice as Cre drivers. Administration of the estrogen analog, tamoxifen (Tam) could induce the translocation of the Cre-ER protein from the cytoplasm to the nucleus; thus, we could express FLAG-GLP in the whole body at any time by Tam treatment. We treated these mice with Tam at 3 to 4 weeks of age (once a week, two times) and confirmed FLAG-GLP expression in more than 85% of both NeuN+ and NeuN- cells by immunostaining of *Ehmt1* iTg; *CAG-CreER* mouse brain at 1 week after the second Tam treatment (Figure S2A). H3K9me2 levels were clearly recovered by the expression of FLAG-GLP at 5 weeks of age (Figure S2B). When we performed behavioral tests at 8–9 weeks of age (4–5 weeks after the Tam treatment), somehow FLAG-GLP-supplied WT mice (iTg) showed a hyperactive phenotype (Figure 1E). In contrast, we did not observe any recovery of abnormal behaviors (hypoactive and anxiety phenotypes) of the *Ehmt1*^Δ/+^ condition by the FLAG-GLP supply (Figure 1E, left panel for total distance and right panel for the rearing number by the open-field test; Figure 1F, for staying time in the light area by the light and dark test). However, the abnormal behaviors improved when we examined these mice again at 16–17 weeks of age (2 months after the Tam treatment) (Figures 1E and 1F). The mean value of the total distance and the rearing number of *Ehmt1*^Δ/+^; iTg with *CAG-CreER* Tg mice were still smaller than those of WT mice (WT vs. Δ/+; iTg), but the difference was not statistically significant (Figure 1E, 16 weeks old). Furthermore, the mean duration of stay in the light area of *Ehmt1*^Δ/+^; iTg with *CAG-CreER* Tg mice was still significantly different from that of WT mice (WT vs. Δ/+; iTg) (p < 0.05), but this was also significantly different from that of the *Ehmt1*^Δ/+^ mice (Δ/+ vs. Δ/+; iTg) (p < 0.05) (Figure 1F, 17 weeks old).

### Postnatal supply of GLP can reverse H3K9me2 levels in post-mitotic neurons of *Ehmt1*^Δ/+^ mice

**Figure 2:**
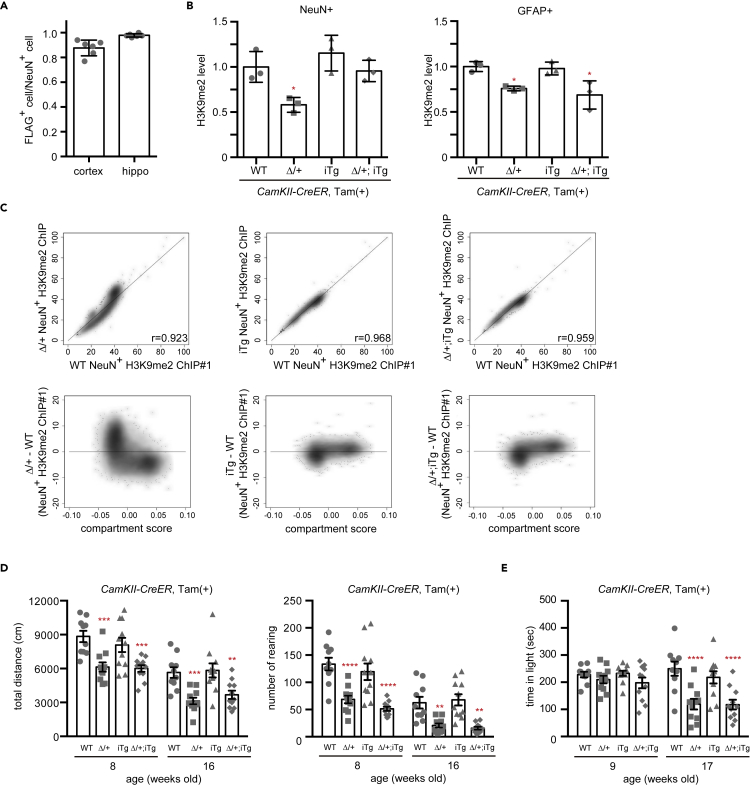
Postnatal neuron-specific GLP supply can reverse diminished H3K9me2 in postmitotic neurons of *Ehmt1*^Δ/+^ mice, but is not enough to rescue abnormal behaviors (A) *Ehmt1* iTg (iTg) with *CamKII-CreER* mice were treated with Tam at 3 and 4 weeks old and dissected at 8 weeks old followed by IHC analysis. Ratio of FLAG+ cells in NeuN+ cells in cortex or hippocampus (hippo) was shown as mean ± SD (n = 6). (B) Wild type (WT, circle), *Ehmt1*^Δ/+^ (Δ/+, square), *Ehmt1* iTg (iTg, up triangle) and *Ehmt1*^Δ/+^; iTg (Δ/+; iTg, diamond) with *CamKII-CreER* mice were treated with Tam at 3 weeks old and dissected at 8 weeks old followed by IHC analysis. H3K9me2 level of NeuN+ or GFAP+ cells (counted 40 cells per animal for NeuN+ or GFAP+ cells, respectively) in the frontal cortex was normalized with pan H3 staining and calculated with the mean of WT as 1. The graph showed individual data for each animal and mean ± SD (n = 3). One-way ANOVA test. ^∗^p < 0.05 (vs WT). (C) H3K9me2 ChIP-seq analysis was carried out using NeuN+ nuclear fraction prepared from WT, Δ/+, iTg and Δ/+; iTg mice cortex (Tam treatment at 3 weeks old, and dissection at 12 weeks old). H3K9me2 ChIP-seq reads per million (RPM) in 80 kb window (Δ/+, iTg or Δ/+; iTg vs WT) are shown and Pearson's R value is stated at the right bottom corner (upper panels). Lower panels show a scatterplot with compartment score on the X axis and H3K9me2 change in each sample on the Y axis. Positive and negative compartment score are defined as A and B compartment. The A compartment was found to be associated with open chromatin and the B compartment with closed chromatin (Lieberman-Aiden et al., 2009). (D) Open field test of wild-type (WT, circle), *Ehmt1*^Δ/+^ (Δ/+, square), *Ehmt1* iTg (iTg, up triangle), and *Ehmt1*^Δ/+^; iTg (Δ/+; iTg, diamond) with *CamKII-CreER* mice treated with Tam at 3 and 4 weeks old (n = 9–12) was examined at 8 or 16 weeks old. Total distance (cm) (left) and the rearing number (right) during 20 min test were shown as mean ± SD. ^∗∗^p < 0.01, ^∗∗∗^p < 0.001 and ^∗∗∗∗^p < 0.0001 (vs WT), repeated two-way ANOVA. (E) Light/dark test was subjected at 9- and 17-week-old mice shown in (D). Staying time in light (sec) was shown as mean + SD. ^∗^p < 0.05 and ^∗∗∗∗^p < 0.0001 (vs WT), repeated two-way ANOVA. See also Figures S2–S4 and S6 and Table S1.

Whole-body postnatal GLP supply from the juvenile stage clearly improved abnormal behavior in *Ehmt1*^Δ/+^ mice. Thus, we next limited the GLP supply only to the forebrain neurons to evaluate whether both neuron and non-neural cells were required for rescue. For this purpose, we used *CamKII-CreER* Tg as a Cre driver, in which the Cre-ER fusion protein is specifically expressed in the postnatal forebrain neurons (Erdmann et al., 2007). We administered Tam at the same timing for the *CAG-CreER* Tg series (3–4 weeks old) and analyzed the expression of FLAG-GLP in the brain at 8 weeks of age. As shown in Figures 2A and S2C, >80% and almost all NeuN+ neurons showed FLAG+ in the cortex and hippocampus of *Ehmt1*^Δ/+^; *Ehmt1 iTg* (Δ/+; iTg) with *CamKII-CreER* Tg mice, respectively. After Tam induction, total GLP amounts (endogenous and FLAG-exogenous) in NeuN+ cells were 1.4–1.6 times more than that in WT but were not affected in GFAP+ cells (Figure S2D). Subsequently, we checked the level of H3K9me2 in *Ehmt1*^Δ/+^ NeuN+ neurons and GFAP+ astrocytes in the frontal cortex of the FLAG-GLP-induced mice at 8 weeks of age (4 weeks after Tam treatment). As shown in Figures 2B and S3A, the H3K9me2 levels in *Ehmt1*^Δ/+^(Δ/+) neurons (NeuN+) and astrocytes (GFAP+) were lower than those in their WT littermates, and FLAG-GLP expression induced by *CamKII-CreER* Tg with Tam increased H3K9me2 signals in the neurons almost similar to the WT level but not in the astrocytes of *Ehmt1*^Δ/+^; *Ehmt1 iTg* mice (Δ/+; iTg). Collectively, these results indicate that GLP (probably a G9a/GLP complex)-mediated H3K9me2 can be induced in terminally differentiated postmitotic neuronal cells.

Although the levels of H3K9me2 in *Ehmt1*^Δ/+^ neurons were reversed by GLP supply, genome-wide H3K9me2 distribution of the rescued *Ehmt1*^Δ/+^ neurons might be different from that of the WT population. Therefore, we induced FLAG-GLP expression with Tam at 3–4 weeks of age, isolated NeuN+ nuclei from the frontal cortex at 12 weeks of age, and performed chromatin immunoprecipitation sequencing (ChIP-seq) analysis. In *Ehmt1*^Δ/+^ NeuN+ nuclei, H3K9me2 levels decreased in the low-to-middle H3K9me2 chromatin regions and increased in the high H3K9me2 regions compared to that in WT NeuN+ nuclei chromatin (Figure 2C upper left panel and Table S1). Furthermore, compartment score analysis showed that decreased H3K9me2 chromatin regions in the *Ehmt1*^Δ/+^ NeuN+ nuclei were in the A compartment (positive compartment score regions), representing euchromatic regions; and that oppositely increased H3K9me2 regions are in the B compartment (negative compartment score regions), showing heterochromatic regions (Figure 2C lower left panel). Moreover, the *Ehmt1*^Δ/+^ H3K9me2 phenotypes were canceled in compound mouse neurons (*Ehmt1*^Δ/+^; *iTg*) by FLAG-GLP expression (Figure 2C, right panels). We repeated the same experiment again (ChIP#2) and confirmed the reproducibility of these results (Figures S3B and S3C and Table S1). In addition, we analyzed the reported H3K9me2 ChIP-seq data of WT and *Ehmt1*^Δ/+^ brain samples and obtained similar results (Figures S4A and S4B) (Iacono et al., 2018). Taken together, we concluded that *Ehmt1* haploinsufficiency induces global reduction of euchromatic H3K9me2 in the brain, and that postnatal supply of GLP can reverse the diminished H3K9me2 phenotype even in a postmitotic situation.

### Forebrain neuron-specific postnatal GLP supply cannot rescue abnormal behaviors of *Ehmt1*^Δ/+^ mice

We then examined whether abnormal behaviors of *Ehmt1*^Δ/+^ mice could be improved by postnatal neuron-specific expression of FLAG-GLP from the juvenile stage. We performed behavioral tests at 8 weeks of age (4 weeks after the Tam injection), which was sufficient to recover the H3K9me2 level in *Ehmt1*^Δ/+^; *iTg* neurons (Figure 2B). As expected from the results of the *CAG-CreER* Tg ubiquitous rescue experiments, low locomotor and exploratory phenotypes observed in *Ehmt1*^Δ/+^ mice were not reversed (Figure 2D). We retested them again at 16 weeks of age, but again, we could not observe any recovery even at 12 weeks after induction. We also assessed the anxiety phenotype in *Ehmt1*^Δ/+^ mice using light and dark test, but the neuron-specific FLAG-GLP supply did not improve the elevated anxiety phenotype observed in *Ehmt1*^Δ/+^ mice (Figure 2E). This suggests that forebrain neuron-specific FLAG-GLP expression is not sufficient, and that GLP supply to additional neural cells other than frontal neuronal cells and non-neural cells is required to improve behavioral phenotypes.

### Increase of activated microglia and upregulation of inflammation-related genes in *Ehmt1*^Δ/+^ mouse brain

**Figure 3:**
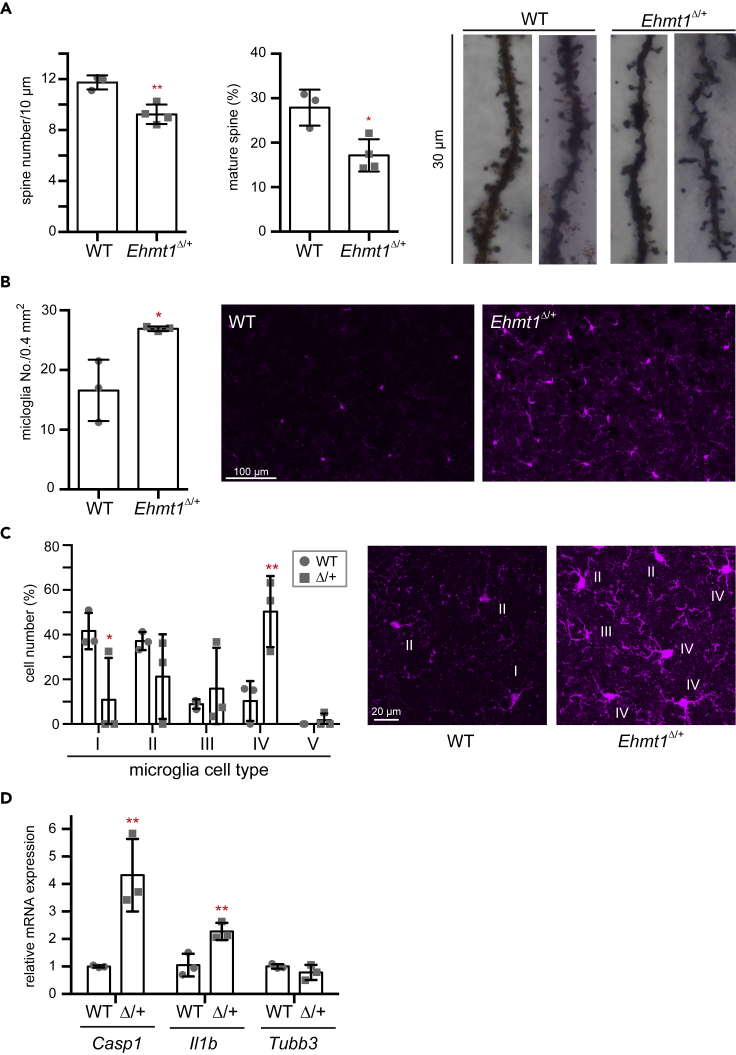
*Ehmt1*^Δ/+^ mice brain shows neuroinflammatory phenotype (A) Golgi staining was performed using 14-week-old WT (circle) and *Ehmt1*^Δ/+^ (square) mouse cortex (n = 3 for WT, n = 4 for *Ehmt1*^Δ/+^ mice). Twenty dendrites selected from independent pyramidal neuron in layer II-III were examined for each animal. The spine number (left) and maturation rate (middle) were shown as individual value and mean ± SD. ^∗^p < 0.05, ^∗∗^p < 0.01 (vs WT), unpaired t test. Mushroom shape (head width > 0.6 μm) and branched shape spines were classified as mature spines (Risher et al., 2014). Images of the representative Golgi staining were shown in right. (B) The number of Iba1+ cells in cortex area (0.4 mm^2^) of WT (circle) and *Ehmt1*^Δ/+^ (square) mice at 7 weeks old was shown as mean ± SD (n = 3). ^∗^p < 0.05 unpaired t test. Images of the representative Iba1 immunostaining (magenta) were shown in right. (C) Iba1+ cells were classified into 5 morphological groups (illustrated in Figure S4C, Diz-Chaves et al., 2012). In brief, type I; cell with fewer dendrites (≤ 2), type II; cell with 4 dendrites, type III; small cell body with numerous dendrites, type IV; large cell body with retracted and thicker dendrites, type V; cells with ameboid cell body, numerous short dendrites. Proportion of cell number (%) were shown as mean ± SD. ^∗^p < 0.05 and ^∗∗^p < 0.01 (vs WT), two-way ANOVA. Iba1 immunostaining (magenta) were shown in right and the classification of microglia was shown with white number by the cell. (D) *Casp1* and *ll1b* mRNA were increased in *Ehmt1*^Δ/+^ mice cortex at 17 weeks old. *Tubb3* (Class III β-tubulin) was used as house-keeping gene control. Relative mRNA expression was calculated by the mean of WT as 1 and plotted with individual values and mean ± SD. n = 3 each, ^∗^p < 0.05, ^∗∗^p < 0.01 unpaired t test. See also Figures S4 and S6.

**Figure 4:**
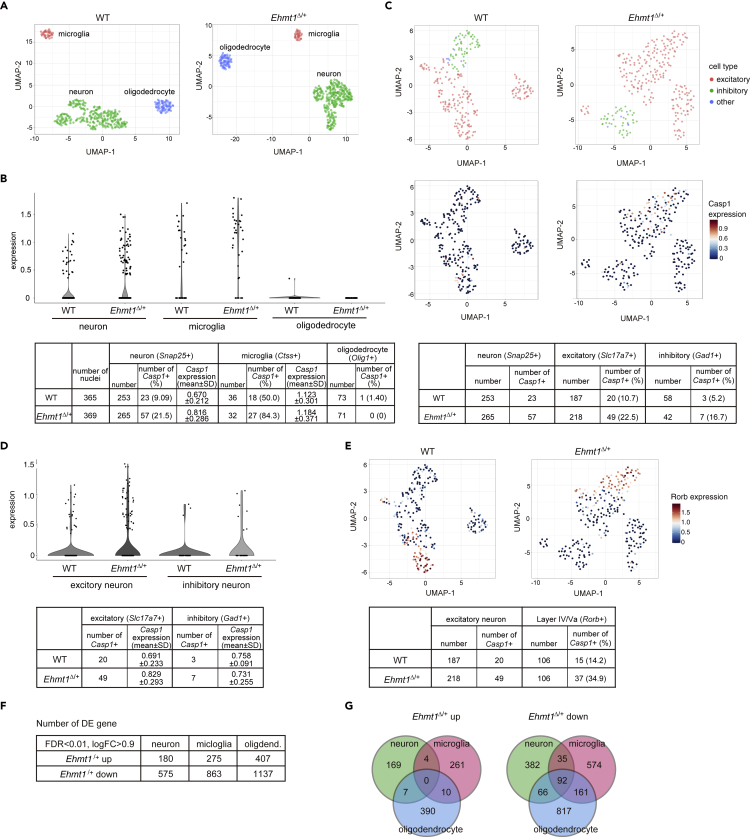
Single nuclei RNA-seq analysis revealed *Casp1* upregulation in neuron and microglia in *Ehmt1*^Δ/+^ mice (A) Single nuclear RNA sequencing of ∼370 nuclei isolated from mouse frontal cortical region using 3 WT or *Ehmt1*^Δ/+^ mice each (14 weeks old). UMAP plots showed the cluster of neuron (green), microglia (pink) and oligodendrocyte (blue), respectively. (B) Comparison of *Casp1* expression in wild type and *Ehmt1*^Δ/+^ mice neuron (*Snap25*+), microglia (*Ctss*+) and oligodendrocyte (*Olig1*+) was shown as a violin plot. Number of *Casp1* expressed cells and mean ± SD of *Casp1* expression in *Casp1*+ cells were summarized in table below the plot. (C) UMAP plot of neural cells isolated from WT or *Ehmt1*^Δ/+^ cortex colored by cell type (top, excitatory neuron; pink, inhibitory neuron; green, other; blue) and *Casp1* expression (bottom). Number of *Casp1* expressed cells among excitatory (*Slc17a7*+) or inhibitory (*GAD1*+) neuron was shown as a table below. (D) Comparison of *Casp1* expression in wild type and *Ehmt1*^Δ/+^ mice excitatory and inhibitory neuron was shown as a violin plot. Number of *Casp1* expressed cells and mean ± SD of *Casp1* expression in *Casp1*+ cells were summarized in a table below the plot. (E) UMAP visualization of *Rorb* expressed neuron in WT or *Ehmt1*^Δ/+^ mice. Table below the plot showed number of *Casp1*+ cell in *Rorb* + population. (F) The number of differentially expressed (DE) genes (FDR < 0.01, logFC > 0.9 compared with wild type) in neurons, microglia and oligodendrocyte of *Ehmt1*^Δ/+^ mouse cortex characterized by single nuclei RNA-seq analysis. (G) Overlap of DE genes among neuron, microglia and oligodendrocyte of *Ehmt1*^Δ/+^ mouse cortex (left panel: upregulated genes; right panel: downregulated genes). See also Figures S5–S7, Tables S2 and S3.

**Figure 5:**
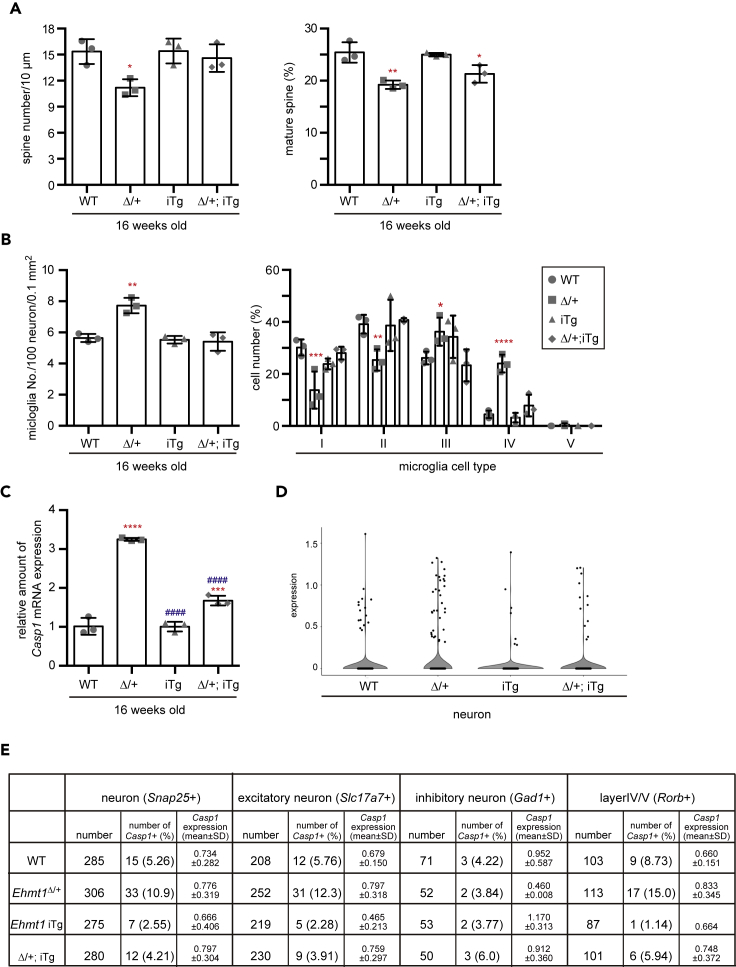
Brain phenotypes in *Ehmt1*^Δ/+^ mice can be reversed by neuron-specific postnatal supply of GLP (A) Golgi staining was performed using 16-week-old mouse cortex of wild type (WT, circle), *Ehmt1*^Δ/+^ (Δ/+, square), *Ehmt1* iTg (iTg, up triangle) and *Ehmt1*^Δ/+^; iTg (Δ/+; iTg, diamond) with *CamKII-CreER* mice treated with Tam at 3 weeks old (n = 3 each). Individual values of spine number (left) and maturation rate (right) and mean ± SD were shown. ^∗^p < 0.05, ^∗∗^p < 0.01 (vs WT), one-way ANOVA. Images of the Golgi staining were shown in Figure S5A. (B) Left, microglia number in cortex area (0.1 mm^2^) at 16 weeks old (Tam injection at 3 weeks old) was normalized with NeuN+ cell number and shown as individual values and mean ± SD. ^∗∗^p < 0.01 (vs WT), One-way ANOVA (n = 3 each). Right, microglias were classified into 5 classed by morphology (same criteria as for Figure 3C). Proportion of cell number (%) were shown as individual values and mean ± SD. ^∗^p < 0.05, ^∗∗^p < 0.01, ^∗∗∗^p < 0.001 and ^∗∗∗∗^p < 0.0001 (vs WT), two-way ANOVA. Iba1 immunostaining (magenta) were shown in Figure S5D. (C) RNA was extracted from cortex of *CamKII-CreER* mouse group at 16 weeks old (Tam injection at 3 and 4 weeks old). *Casp1* mRNA was measured and relative expression level was shown as mean ± SD. ^∗∗∗^p < 0.001, ^∗∗∗∗^p < 0.0001 (vs WT) and ^####^p < 0.0001 (iTg or Δ/+; iTg vs *Ehmt1*^Δ/+^), One-way ANOVA. (D) Expression of *Casp1* in neuron was analyzed by single nuclei RNAseq prepared from cortex of wild type, *Ehmt1*^Δ/+^, *Ehmt1* iTg and *Ehmt1*^Δ/+^; iTg mice with *CamKII-CreER. Casp1* expression was shown as a violin plot (top). (E) *Casp1* expression in neuron (*Snap25*+), excitatory neuron (*Slc17a7*+), inhibitory neuron (*GAD1*+) and layer IV/V neuron (*Rorb1*+) was analyzed by single nuclei RNAseq. Number of *Casp1* expressed cells and mean ± SD of *Casp1* expression in *Casp1*+ cells were summarized in a table. See also Figure S7.

In addition to behavioral abnormalities, it has been reported that CA1 hippocampal neurons in *Ehmt1*^Δ/+^ mice show a decrease in their number of mature spines (Balemans et al., 2013). We also confirmed that spine density of frontal cortex neurons decreased (Figure 3A left) and that the ratio of mature spines in *Ehmt1*^Δ/+^ mice was diminished compared to that in the WT mice (Figure 3A right). Furthermore, by inspection of neural and non-neural populations in *Ehmt1*^Δ/+^ mouse brain sections, we discovered that the number of Iba1^+^ microglial cells significantly increased in the *Ehmt1*^Δ/+^ mouse cortical brain (Figure 3B). The active state of microglia is classified by morphology (Diz-Chaves et al., 2012). We found that the ratio of class IV type cells, which is a more activated form, increased, and that the ratio of inactive class I/II type cells decreased in *Ehmt1*^Δ/+^ mice, indicating that activated microglial cells increased in the *Ehmt1*^Δ/+^ mouse brain (Figures 3C and S4C). Consistent with the increase in activated microglial cells in the *Ehmt1*^Δ/+^ brain, some inflammation-related genes, including *Caspase-1* (*Casp1*) and *IL-1β (Il1b)* mRNA expression, were upregulated several times in the *Ehmt1*^Δ/+^ brain (Figure 3D). Indeed, upregulation of *Casp1* and *Il1b* mRNA has been observed in forebrain neuron-specific *Ehmt1* or *Ehmt2* KO mouse brains, especially in the cortex region (Schaefer et al., 2009). Furthermore, we performed single-nucleus RNA-seq analysis using the frontal cortex regions of WT and *Ehmt1*^Δ/+^ mice (Figure 4A). For this analysis, we utilized our method of single RNA-seq with random displacement amplification sequencing (RamDA-seq), which has high sensitivity with wide-range quantitative linearity and near-complete full-length transcript coverage (Hayashi et al., 2018). We found that *Casp1* is expressed in neurons (*Snap25*+) and microglia (*Ctss*+) in the WT brain, and that the *Casp1*+ population in both cell types increased in *the Ehmt1*^Δ/+^ brain (Figure 4B, WT 9.09% vs. *Ehmt1*^Δ/+^ 21.5% in the neurons and WT 50.0% vs *Ehmt1*^Δ/+^ 84.3% in the microglia, respectively). The amount of *Casp1* transcripts among the *Casp1*+ neurons, but not microglia, slightly increased, but this difference was not statistically significant (Figure 4B lower panel, WT 0.670 ± 0.212 vs. *Ehmt1*^Δ/+^ 0.816 ± 0.286 in the neurons and WT 1.123 ± 0.301 vs. *Ehmt1*^Δ/+^ 1.184 ± 0.371 in the microglia, respectively). We examined the nature of *Casp1*+ cells in the neuronal population of WT and *Ehmt1*^Δ/+^ brains. As shown in Figure 4C, the majority of *Casp1*+ cells (20/23 in WT and 49/57 in *Ehmt1*^Δ/+^) were excitatory neurons (*Slc17a7*+). The amount of *Casp1* transcripts among the *Casp1*+ excitatory neurons slightly increased in *the Ehmt1*^Δ/+^ brain; however, this difference was also not statistically significant (Figure 4D). We further examined which layer neurons among the excitatory neurons were *Casp1*+ and found that the majority of *Casp1*+ excitatory neurons (15/20) were layer 4/5a specific marker gene *Rorb*+ in WT, and that the ratio of *Casp1*+ cells among the *Rorb*^+^ excitatory neurons increased in the *Ehmt1*^Δ/+^ brain (14.2% in WT vs. 34.9% in *Ehmt1*^Δ/+^) (Figures 4C–4E). This trend was also reproduced in another set of single nucleus RNA-seq analyses (Figures 5D and 5E). Thus, layer-specific excitatory neurons may play a role in inflammation-associated phenotypes.

Single-nucleus RNA-seq analysis also showed that many genes were up or downregulated (FDR < 0.01, logFC > 0.9) in different cell types (neurons, microglia, and oligodendrocytes) of *the Ehmt1*^Δ/+^ brain (Figure 4F and Table S2). The upregulated genes of each cell type were mostly unique, but a proportion of the downregulated genes overlapped among them (Figure 4G). Interestingly, Gene Ontology (GO) term enrichment analysis showed that GO terms related to immune response or cytokine production were enriched in the upregulated genes of *Ehmt1*^Δ/+^ neuronal cells (Table S3). This suggests that the accumulation of activated microglial cells in *the Ehmt1*^Δ/+^ brain is caused by the upregulation of immune responsive genes in neurons.

### Neurological phenotypes of *Ehmt1*^Δ/+^ mice were reversed by neuron-specific postnatal supply of GLP

We then investigated whether forebrain neuron-specific postnatal GLP supply had any impact on *Ehmt1*^Δ/+^ brain pathological phenotypes, although behavioral abnormalities were not recovered. Strikingly, the reduced number of spines (Figure 5A left and Figure S5A) and the increased number of microglial cells (Figure 5B left) in *the Ehmt1*^Δ/+^ brain were both reinstated to the WT number by the neuron-specific postnatal GLP supply from 3 weeks of age. Furthermore, increased *Casp1* expression in *the Ehmt1*^Δ/+^ brain was also strongly restored by the induction of FLAG-GLP expression (Figure 5C). We also confirmed that *Casp1* expression and the *Casp1*+ population in neurons were decreased by single nucleus RNA-seq analysis (Figures 5D, 5E, and S5B). Consistently, the level of H3K9me2 in the promoter region of *Casp1* was reduced in *Ehmt1*^Δ/+^ NeuN+ neuronal cells, which was reverted to the WT level by FLAG-GLP expression (Figure S5C). Although the activated morphology of microglia was normalized by FLAG-GLP expression (Figure 5B right and Figure S5D), spine morphology was not reversed (Figure 5A right). The dendritic shafts of *Ehmt1*^Δ/+^; iTg mice were still immature and thin, and the population of mature spines was still decreased, as seen in *Ehmt1*^Δ/+^ mice (Figure 5A right and Figure S5A). Although postnatal GLP supply in the neurons was not sufficient to rescue abnormal behaviors as described above, the results demonstrated that some of the *Ehmt1*^Δ/+^ brain phenotypes are reversible by postnatal GLP supply only in neurons. Furthermore, we showed that the accumulation of microglial cells in the *Ehmt1*^Δ/+^ brain is induced by insufficient expression of GLP in the neurons.

### The IL-1β pathway is involved in the neurological phenotypes of *Ehmt1*^Δ/+^ mice

**Figure 6:**
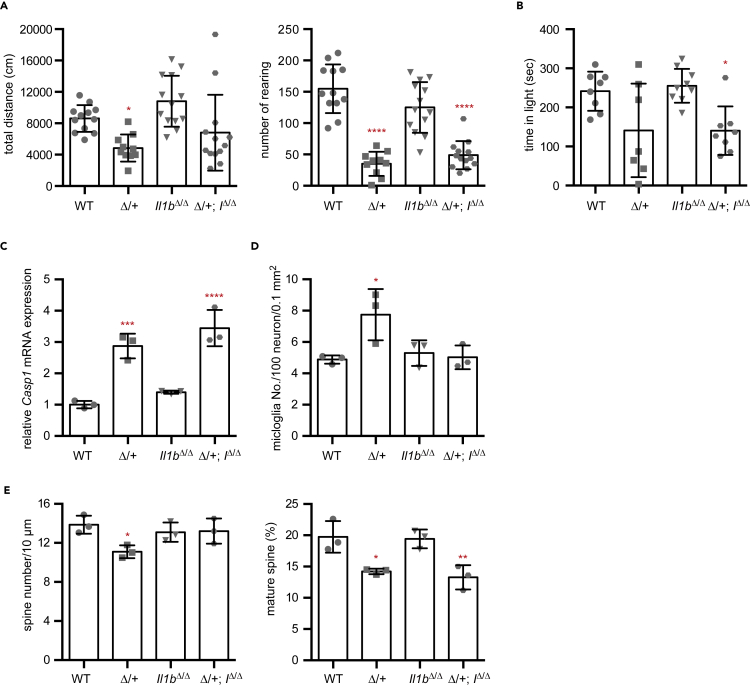
Depletion of IL-1β can rescue neurological phenotypes but not behavioral phenotypes of *Ehmt1*^Δ/+^ mice (A) Open field test of wild type (WT, circle), *Ehmt1*^Δ/+^ (Δ/+, square), *Il1b*^Δ/Δ^ (down triangle), and *Ehmt1*^Δ/+^; *Il1b*^Δ/Δ^ (Δ/+; *I*^Δ/Δ^, hexagon) (n = 10–13) was examined at 12 weeks old. Total distance (cm) (left) and rearing number (right) were shown as individual values and mean ± SD. ^∗^p < 0.05, ^∗∗∗∗^p < 0.0001 (vs WT), one-way ANOVA. (B) Light/dark test of *Ehmt1*^Δ/+^ and *Il1b*^Δ/Δ^ mouse group was analyzed at 12 weeks old (n = 10–13). Staying time in light (sec) was shown as individual values and mean ± SD. ^∗^p < 0.05 (vs WT), One-way ANOVA. (C) RNA was extracted from mice cortex of wild type (WT, gray), *Ehmt1*^Δ/+^ (Δ/+, light gray), *Il1b*^Δ/Δ^ (black), and *Ehmt1*^Δ/+^; *Il1b*^Δ/Δ^ (Δ/+; *I*^Δ/Δ^, grid line) at 13 weeks old (n = 3). Relative expression level of *Casp1* mRNA was shown as individual value mean ± SD (the mean of WT as 1). ^∗∗∗^p < 0.001, ^∗∗∗∗^p < 0.0001 (vs WT), One-way ANOVA. (D) Microglia number in cortex area (0.1 mm^2^) at 13 weeks old mice (n = 3 each) was normalized with NeuN+ cell number and shown as mean ± SD. ^∗^p < 0.05 (vs WT), One-way ANOVA. (E) Golgi staining was performed using 13-week-old mouse brain (n = 3 each). Twenty five dendrites chosen from layerII-III pyramidal neuron at cortical region were examined for each animal and spine number (left) and maturation rate (right) were shown as individual values and mean ± SD. ^∗^p < 0.05, ^∗∗^p < 0.01 (vs WT), One-way ANOVA.

Postnatal GLP supply in neurons can suppress *Casp1* gene expression and reverse microglial activation in *Ehmt1*^Δ/+^ mice, which led us to hypothesize that the inflammatory response is involved in a part of KS phenotypes induced by *Ehmt1* haploinsufficiency. Since Caspase-1 activates IL-1β, we introduced *Il1b* deficiency in *Ehmt1*^Δ/+^ mice to evaluate the role of upregulated expression of *Casp1* and *Il1b* in the neurological and behavioral phenotypes of *Ehmt1*^Δ/+^ mice. We performed behavioral analyses using these compound mice. IL-1β administration was reported to increase anxiety in WT mice (Li et al., 2018); thus, we hypothesized that *Il1b* KO might rescue the behavioral abnormalities observed in *Ehmt1*^Δ/+^ mice. Contrary to our assumption, the locomotor activity of *Ehmt1*^Δ/+^; *Il1b*^Δ/Δ^ mice was partially increased compared to that of *Ehmt1*^Δ/+^ mice (Figure 6A left, total distance) but did not fully recover to the wild-type level. In addition, the anxiety phenotype was not rescued by *Il1b* KO mice (Figures 6A right and 6B). We next examined the neurological phenotypes of these compound mice. Caspase-1 is an upstream of IL-1β, thus, *Casp1* expression was still elevated in compound mice as *Ehmt1*^Δ/+^ mice (Figure 6C). However, increased microglia numbers were restored by *Il1b* KO (*Il1b*^Δ/Δ^) in *Ehmt1*^Δ/+^ mice (Figure 6D), suggesting that activation of the IL-1β pathway induced microglial activation in *Ehmt1*^Δ/+^ mice. Although the spine number was restored by *Il1b* KO (*Il1b*^Δ/Δ^) in *Ehmt1*^Δ/+^ mice (Figure 6E left), as expected from recovery of microglia number, the maturation rate of the spine remained low in the compound mice (Figure 6E right). Therefore, although the spine number was increased, these spines seemed to be immature to construct proper neural networks in *Ehmt1*^Δ/+^; *Il1b*^Δ/Δ^ mice, which may be insufficient for reversal of behavioral abnormalities. From these data, we showed that *Ehmt1* haploinsufficiency activates the IL-1β microglia pathway and that the IL-1β pathway is involved in some *Ehmt1*^Δ/+^ neurological phenotypes, but that *Il1b* KO alone was not sufficient to rescue abnormal behavior in *Ehmt1*^Δ/+^ mice. This further indicated that *Ehmt1*^Δ/+^ behavioral abnormalities were composed of malfunctions of IL-1β and other pathways

## Discussion

In this study, we showed that (1) abnormal behaviors of *Ehmt1*^Δ/+^ mice are difficult to treat from the postnatal stage but can be improved if GLP is ubiquitously supplied from the juvenile stage (most likely if supplied to whole brain cells); (2) postnatal GLP supply can induce the upregulation of H3K9me2 in neurons such as terminally differentiated postmitotic cells; (3) neurological phenotypes of *Ehmt1*^Δ/+^ mice, such as a reduction in the number of mature spines and accumulation of activated microglia, can be reversed by GLP supply even after birth; and (4) the IL-1β pathway is responsible for some of the neurological phenotypes of *Ehmt1*^Δ/+^ mice.

From different GLP supply experiments, we observed that KS-like neurological phenotypes were suppressed when GLP was specifically supplied to both neural and non-neural brain cells from the neonatal stage. Postnatal GLP supply also improved but did not fully reverse the phenotypes, indicating that it is better to treat the KS model at an earlier stage (Figure 1). In addition, it took time to improve the abnormal behavior in the postnatal GLP supply (Figures 1E and 1F). This suggests that it progresses slowly during recovery of neural network formation. Similar postnatal rescue experiments of autism-related mouse models, including *MeCP2* (Guy et al., 2007; Sztainberg et al., 2015), *Ube3a* (Silva-Santos et al., 2015), *Syngap1* (Clement et al., 2012), and *Shank3* (Mei et al., 2016) mutants or duplications have been reported. Similar to the KS model studies, recovery of genes responsible for the *CAG-CreER* Tg system can reverse cellular functions such as spine density and synaptic transmission potential over a wide range of postnatal neurodevelopmental windows, but abnormal behavioral or cognitive phenotypes such as anxiety and motor coordination were hardly reversed after the critical time window, especially during the adolescent stage (Clement et al., 2012; Guy et al., 2007; Mei et al., 2016; Silva-Santos et al., 2015; Sztainberg et al., 2015). Rett syndrome is now considered a possible treatable disorder based on the reversibility of neurological defects in *Mecp2*-deficient Rett syndrome model mice by the postnatal activation of MeCP2 expression (Guy et al., 2007; Lioy et al., 2011; Vashi and Justice, 2019). A previous *Drosophila* study has shown that cognitive defects induced by *EHMT* mutation can be restored by *EHMT* re-expression during adulthood (Kramer et al., 2011), suggesting that defects in neural functions induced by *EHMT* deficiency can be postnatally fixed. Our new findings further encourage the possibility that KS phenotypes, including certain behavioral abnormalities, can be suppressed or reversed if GLP expression or function is complemented by a critical neurodevelopmental time window even after birth.

Epigenetic information is maintained beyond cell division to maintain its unique cellular nature, whereas it is also known to be plastically affected by internal or external factors. However, it is not clear whether epigenetic manipulation is effective in postnatal neurons since the majority of neurons are post-mitotic, such as in the G0-G1 stage. Our recent study showed that G9a-dependent H3K9me2 can be induced in the G1 phase of the cell cycle (Fukuda et al., 2019), but *the in vivo* situation in post-mitotic cells has not been clarified. Upregulation of H3K9me2 immunostaining signals in NeuN^+^ neurons is induced in mice after 3 days of light deprivation (Benevento et al., 2016), suggesting that H3K9me2 is also plastic in post-mitotic cells *in vivo*. Our new data further support this, which means that H3K9me2 and its functions in postnatal neurons can be a target for epigenome manipulation *in vivo*. It has been reported that more than 660 genes are thought to cause human disease as a result of haploinsufficiency and multiple histone methyltransferases or demethylase genes other than *EHMT1*, such as *KMT2A*, *NSD1, KDM5C*, and *KDM6A* are included in them (Matharu et al., 2019). Haploinsufficiency of such epigenome-regulating genes show mental retardation or autistic-like features in most cases (https://medlineplus.gov/genetics/gene/). Postnatal epigenome manipulation may be applicable to impaired neural function in haploinsufficiency diseases of histone methylation-regulating genes.

H3K9me2 reduction in the *Ehmt1*^Δ/+^ mouse brain altered gene expression. We showed that the inflammatory gene, *Casp1* mRNA, is upregulated in *Ehmt1*^Δ/+^ mouse neurons and microglia (Figure 4B). We also detected an increase in *Il1b* mRNA, which is a downstream factor of Caspase-1 (Figure 3D). Caspase-1 forms an inflammasome together with NLRP3 and the complex induces pro-inflammatory cytokine IL-1β, suggesting that *Ehmt1*^Δ/+^ mouse brains are under neuroinflammation. GO analysis of single nuclear RNA-seq showed upregulation of immune response or cytokine production, supporting this idea (Table S3). The Caspase-1-IL-1β signaling pathway is known to induce the proliferation and activation of microglial cells in the brain. We showed that the number of microglial cells, especially activated cells, increased in the *Ehmt1*^Δ/+^ mouse brain (Figures 3B and 3C). Microglia are involved not only in immune responses but also in normal brain development (Prinz et al., 2019). It mediates spine maturation and neuronal circuit formation through a spine-pruning function (Paolicelli et al., 2011). This suggests that decreased spine density and maturation in *Ehmt1*^Δ/+^ mice were caused by activated spine-pruning function (Figure 3A). We found that type IV microglial cells, which are active for pruning but not for phagocytosis, increased significantly in *the Ehmt1*^Δ/+^ brain (Figure 3C), thus supporting our hypothesis. Interestingly, postnatal supply of GLP specific to neurons reversed not only upregulation of *Casp1* expression but also microglia and spine phenotypes in *Ehmt1*^Δ/+^ mice (Figure 5). This indicates that inhibition of inflammation in neurons is sufficient to block microglial activation and spine number reduction. Based on our new findings and published data, we propose a possible pathological mechanism for *Ehmt1*^Δ/+^ mice. Reduction of the G9a/GLP complex in *Ehmt1*^Δ/+^ neuronal cells diminishes H3K9me2 at the global level, including *the Casp1* locus (Figure S5C); while *Casp1* expression was upregulated in neurons (Figures 3D and 4B). This upregulation of Caspase-1 activates the Caspase-1-IL-1β signaling inflammasome pathway, which is followed by microglial activation. Activated microglia induce excess pruning of the synaptic spine, thus leading to the disruption of the neural network. Finally, this may result in abnormal behavior in *Ehmt1*^Δ/+^ mice. Dysfunction of microglia has been reported to be involved in several neurodevelopmental diseases, including autism spectrum disorder, schizophrenia, and Rett syndrome (Cronk et al., 2015; Sekar et al., 2016). In agreement with our data, overproduction of Caspase-1 in the hippocampus or intracerebroventricular administration of IL-1β induces depression- and anxiety-like behaviors in mice (Li et al., 2018). To elucidate the relationship between the IL-1β pathway and microglia, we further depleted IL-1β in the *Ehmt1*^Δ/+^ background. As expected, *Il1b* KO rescued *Ehmt1*^Δ/+^ microglial activation and spine phenotypes. However, it was not sufficient to rescue abnormal behavior, suggesting that other pathways are also involved in *Ehmt1*^Δ/+^ behavioral phenotypes. A recent report showed that NMDA receptor-dependent neural network activity is altered in neurons induced from iPS cells of patients with KS and in *Ehmt1*^Δ/+^ mice, and that this neural network phenotype can be rescued by NMDA receptor antagonists (Frega et al., 2019). Furthermore, G9a/GLP mediates *BDNF* repression and regulates synaptic scaling *in vivo* and *in vitro* (Benevento et al., 2016). These NMDA receptor or BDNF pathways are also required for the full recovery of abnormal behavior in *Ehmt1*^Δ/+^ mice.

In conclusion, we demonstrated that some of the *Ehmt1*^Δ/+^ neurological phenotypes were reversible, even after birth, by GLP supply. *Ehmt1* haploinsufficiency activates neuroinflammation induced by the Caspase-1-IL-1β pathway. Thus, these data suggest that the IL-1β pathway is a novel potential therapeutic target for the treatment if activation of the Caspase-1-IL-1b pathway is also induced in KS.

### Limitations of the study

H3K9me2 and some of neurological phenotypes including abnormal behaviors were reversed by post-natal supply of GLP in the *Ehmt1*^Δ/+^ mice, but it is not known how the findings of this study can be applicable to KS. It is also not known whether the inflammatory genes are upregulated in neuronal cells in KS. Furthermore, postnatal neuron-specific GLP supply was not sufficient for the improvement of abnormal behaviors, suggesting that abnormal behaviors seen in the *Ehmt1*^Δ/+^ mice were caused by insufficient expression of GLP in both neuronal and non-neuronal cells, but which non-neuronal cells were critical remain to be known.

## STAR★METHODS

### Key resources table

**Table:** 

REAGENT or RESOURCE	SOURCE	IDENTIFIER
**Antibodies**		

Mouse anti-Histone H3 K9 dimethyl monoclonal antibody, clone#6D11	Hayashi-Takanaka et al., 2009	N/A
Rabbit anti-Histone H3, CT, pan polyclonal antibody	Millipore	Cat# 07-690; RRID:AB_417398
Mouse anti-GLP monoclonal antibody (B0422)	Tachibana et al., 2005	N/A
Mouse anti-G9a monoclonal antibody (A8620)	Tachibana et al., 2005	N/A
Monoclonal ANTI-FLAG M2 antibody	Sigma-Aldrich	Cat# F3165; RRID:AB_259529
Chicken anti-NeuN polyclonal antibody	Millipore	Cat# ABN91; RRID:AB_11205760
Rabbit anti-Iba1 polyclonal antibody	Wako	Cat# 019-19741; RRID:AB_839504
Chicken anti-GFAP polyclonal Antibody	Abcam	Cat# ab4674; RRID:AB_304558
Goat anti-Mouse IgG (H+L) Cross-Adsorbed Secondary Antibody, Alexa Fluor 488	Thermo Fisher Scientific	Cat# A-11001; RRID:AB_2534069
Goat anti-Rabbit IgG (H+L) Cross-Adsorbed Secondary Antibody, Alexa Fluor 568	Thermo Fisher Scientific	Cat# A-11011; RRID:AB_143157
Goat anti-Chicken IgY (H+L) Secondary Antibody, Alexa Fluor 647	Thermo Fisher Scientific	Cat# A-21449; RRID:AB_2535866
Anti-NeuN, clone A60, Alexa Fluor 488 conjugated antibody	Millipore	Cat# MAB377X; RRID:AB_2149209

**Critical Commercial Assays**		

FD Rapid Golgi Stain Kit	FD Neuro Technologies	Cat# PK401
Sepasol -RNAⅠSuper G	Nacalai Tesque	Cat# 09379-84
Omniscript RT Kit	Qiagen	Cat# 205111
Power SYBR Green PCR Master Mix	Thermo Fisher Scientific	Cat#4367659
KAPA Hyper Prep Kit	Kapa Biosystems	Cat#KK8502

**Deposited Data**		

H3K9me2 ChIP-seq data	This paper	GSE162327
H3K9me2 ChIP-seq data	Iacono et al., 2018	GSE89010
Single nucleus RNA-seq analysis	This paper	GSE162934

**Experimental Models: Organisms/Strains**		

*Ehmt1* heterozygous knockout (*Ehmt1*^Δ/+^) mouse (C57BL6/J)	Tachibana et al., 2005	N/A
*Rosa26-CAG-FLAG-Ehmt1* Tg (*Ehmt1* iTg) mouse (C57BL6/J)	Deguchi et al., 2013	N/A
*Nestin-Cre* Tg mouse (C57BL6/J)	Isaka et al., 1999	N/A
B6.Cg-Tg(creEsr1)5AmcJ (*CAG-CreER* Tg) mouse	The Jackson Laboratory	Stock No: 004682
*CamKII-CreER* Tg mouse (C57BL6/J)	Erdmann et al., 2007	N/A
*Il1b* knockout (*Il1b*^Δ/Δ^) mouse, (C57BL6/J)	Horai et al., 1998	N/A

**Oligonucleotides**		

Primers sequences for RT-qPCR and ChIP-qPCR are listed in Table S4		

**Software and Algorithms**		

Prism6	Graphpad	https://www.graphpad.com/
ImageJ	NIH	https://imagej.nih.gov/ij/
R(3.3.3 or 3.6.0)	R Core Team (2017)	https://www.r-project.org/
edgeR (3.22.0)	Robinson et al., 2010	http://bioinf.wehi.edu.au/edgeR/
umap (0.2.7.0)	Leland M et al., 2018	https://github.com/tkonopka/umap
Fastq-mcf (1.04.807)	Aronesty (2011)	https://expressionanalysis.github.io/ea-utils/
FastQC (0.11.5).	Andrews et al. (2010)	https://www.bioinformatics.babraham.ac.uk/projects/fastqc/
HISAT2 (2.0.4)	Kim et al., 2019	http://daehwankimlab.github.io/hisat2/
RSeQC (2.6.4)	Wang et al., 2012	http://rseqc.sourceforge.net/
featureCounts (1.5.0)	Liao et al., 2014	http://subread.sourceforge.net/
Trim Galore (0.3.7)		http://www.bioinformatics.babraham.ac.uk/projects/trim_galore/
Bowtie (4.4.6)	Langmead and Salzberg, 2012	https://doi.org/10.1186/gb-2009-10-3-r25
GSEA		http://software.broadinstitute.org/gsea/

**Other**		

RamDA-seq reagents	Hayashi et al., 2018	N/A

### Resource availability

#### Lead contact

Further information and requests for resources and reagents should be directed to and will be fulfilled by the lead contact, Yoichi Shinkai (yshinkai@riken.jp)

#### Materials availability

All unique reagents generated in this study are available from the Lead Contact with a completed Material Transfer Agreement.

#### Data and code availability

RNA-seq and ChIP-seq data generated during this study have been deposited to NCBI: GSE162934 and GSE162327, respectively.

### Experimental model and subject details

#### Animals

Mice used in this study were under C57BL6/J background and housed as a group (4/cage) in 12 hours light/dark cycle. *Ehmt1*^Δ/+^, *Nestin-Cre*, *CAG-CreER* and *CamKII-CreER* mice were backcrossed with C57BL6/J by the speed congenic method using microsatellite PCR (Ogonuki et al., 2009). All mice experiments were approved by RIKEN safety divisions and conducted under institute rules and regulations. Tamoxifen (Merk) was given by oral administration (100 mg/kg, 2 times, 1 week-interval). Only male mice were used in this study and age of mice for behavioral analysis, Tamoxifen administration and dissection were summarized in Figure S6 and Table S6.

### Method details

#### Behavioral analysis

Male mice (>10) were used for behavioral analysis. At least 1 week-interval was given to mice between behavioral analysis.

1. Open field test

Single mouse was placed in a box (50 cm W X 50 cm L X 40 cm H, 70 lux) for 20 min. Behaviors including traveling distance and rearing number were recorded automatically and analyzed with its software (O’HARA & CO., LTD).

2. Light and dark test

Anxiety was examined using light (200 lux) and dark test for 10 min (O’HARA & CO., LTD). Time and distance in light or dark area, transition number between two areas and time for first latency entered to light area were recorded automatically.

#### Immuno-histological analysis

Brain was dissected out from mice anesthetized and perfused with phosphate buffered saline. Brain slices were soaked in 4% PFA overnight and mounted with O.C.T compound (Sakura Finetek) after 30% sucrose replacement to make frozen section. Antibodies used for IHC were; anti-Histone H3K9me2 (1:500 dil., clone#6D11 (Hayashi-Takanaka et al., 2009)), pan-Histone H3 CT (1:2000 dil., Millipore #07-690), anti-GLP (1:500 dil., clone#0422 (Tachibana et al., 2005)), anti-FLAG (1:2000, Sigma #F3165), anti-NeuN (1:2000 dil., Millipore #ABN91), anti-Iba1 (1:1000 dil., Wako #019-19741) and anti-GFAP (1:1000 dil., abcam #ab4674). Alexa Fluor 488, 564 or 647 conjugated antibodies were used as secondary antibodies (Thermo). Fluorescence images were captured with FV3000 (Olympus) or TCS sp8 (Leica) and intensity of fluorescence was quantitated with ImageJ software.

#### Western blot analysis

Total protein was extracted from whole brain lysed with RIPA buffer (50 mM Tris-HCl (pH7.5), 420 mM NaCl, 1% NP-40, 0.25% sodium deoxycholate, 1 mM EDTA). Twenty μg (for Histone) and 40 μg lysate (for mGLP/G9a) was subjected on SDS-PAGE and blotted with antibodies (anti-HistoneH3 K9me2, pan-Histone H3, mGLP and G9a). Blots were analyzed and quantitated with Odyssey CLx (LI-COR) or ImageJ. Quantified protein band were normalized with internal control (pan Histone H3 or α-Tubulin) and relative amount of proteins was calculated by mean of wild type as 1.

#### Golgi staining

Mouse brain was dissected out and proceeded to Golgi staining with FD Rapid Golgi Stain Kit (FD Neuro Technologies). Images of cortical neuron were taken by Aperio CS2 (Leica) or BX53 (Olympus) and analyzed by Aperio Image Scope (Leica) or ImageJ software. We selected 20-25 pyramidal neurons in layer II-III of cortical region and analyzed spine number on 2^nd^ or 3^rd^ apical dendrite brunch. We only counted spine number from single brunch in one neuron (number of counted dendrites was listed in Table S6). For spine morphology analysis, images were taken by BX53 and analyzed with ImageJ software. Dendritic spines were classified into mature or not mature spine by their shape and head size (Risher et al., 2014). In brief, mushroom shape (head width >0.6 μm) and branched shape spines were classified as mature spines.

#### RT-qPCR analysis

Total RNA was extracted from mice frontal cortex using Sepasol RNA I SuperG (Nacalai). Using 1 μg RNA, reverse transcription was carried with Omniscript RT kit (Qiagen). Quantitative PCR was performed using Power SYBR Green master mix (Thermo) with primers listed in Table S4.

#### Single nucleus RNA-seq analysis

Cell nuclei were isolated from cortical region of adult mice brain (14 weeks old, 3 mice for each genotype were used) as described previously (Mo et al., 2015). In brief, minced cortex was homogenized with ice-cold homogenization buffer (0.25M sucrose, 25 mM KCl, 5 mM MgCl_2_, 20 mM Tricine-KOH (pH7.5), 1mM DTT, 0.15 mM spermine, 0.5 mM spermidine and EDTA-free protease inhibitor) using a loose pestle. After that, IGEPAL-630 was added to homogenate (final conc. 0.3%) and the homogenate was further dounced with a tight pestle. The homogenate was filtered through a 40 μm strainer (Falcon) and mixed with same amount of 50% iodixanol solution (OptiPrep, AXIS-SHIELD) in homogenization buffer. The sample was underplayed with iodixanol gradient (30 and 40%) and centrifuged at 10,000*g* for 18 min in a swinging bucket (SW55Ti, Beckman) at 4°C. Nuclei fraction was placed at the boundary between 30 and 40% iodixanol layer. Collected nuclei were stained with 4′,6-diamidino-2-phenylindole (DAPI, 0.5 μg/ml). Subsequently, single-nucleus sorting was performed using MoFlo Astrios (Beckman Coulter). To remove debris from nucleus suspension, DAPI positive single nuclei were sorted into 1 μl of RamDA-seq cell lysis buffer in a 96-well PCR plate (BIOplastics) or a 384-well PCR plate (Eppendorf). Single-nucleus RNA sequencing libraries were prepared according to the RamDA-seq method (Hayashi et al., 2018). The RamDA-seq libraries were sequenced using Illumina NextSeq 500 (single-read 76 cycle sequencing).

Fastq-mcf (version 1.04.807) was used to trim adapter sequences and generate read lengths of 75 nucleotides (nt) with the parameters “-L 42 -l 42 -k 4 -q 30 -S”. Adaptor trimming and quality assessment of the FASTQ files were performed using Fastq-mcf (version 1.04.807) and FastQC (version 0.11.5). The trimmed reads were mapped to the mouse genome (mm10) using HISAT2 (version 2.0.4) and categorized as exonic, intronic or intergenic using RSeQC (version 2.6.4). In addition, we also executed a variety of quality control to detect poorly performing samples using a combination of RSeQC and the featureCounts function of Subread (version 1.5.0) based on the ratio of reads mapping to mitochondrial RNAs and rRNA (< or > mean±3SD), the coverage profile along gene bodies and number of assigned reads to gene regions (Figure S7A). As a result of quality control, 736 cells (WT, 368 cells; *Ehmt1*^Δ/+^, 368 cells) were used for the following analysis. Raw count values for each gene were quantified using the featureCount with default parameters and Gencode mouse gene annotation (v15).

Further bioinformatics analysis, statistics and data visualization were mainly performed using R. Using the umap R Package (version 0.2.7.0), we performed uniform manifold approximation and projection (UMAP) and identified three clusters showing different features. In addition, some cells with weak cell type characteristics were excluded from the analysis. The final cell types in each cluster were determined using a combination of marker genes identified from literature (Table S5). The final number of cells analyzed was 730 cells (WT Neuron, 253 cells; WT Oligodendrocyte, 73 cells; WT Microglia, 36 cells; *Ehmt1*^Δ/+^ Neuron, 265 cells; *Ehmt1*^Δ/+^ Oligodendrocyte, 71 cells; *Ehmt1*^Δ/+^ Microglia, 32 cells;). Differential expression between wild type and KO mouse is calculated by edgeR (version 3.22.0). Nuclear RNA in neural cells prepared from neuron-specific post-natal mGLP supply series was examined in a similar manner, except poor performing samples were excluded based on the coverage profile along to gene bodies (WT 285 cells, *Ehmt1*^Δ/+^ 306 cells, *Ehmt1* iTg 275 cells, *Ehmt1*^Δ/+^; iTg 285 cells) (Figure S7B).

#### ChIP-seq analysis

For ChIP-seq analysis, nuclei were isolated from frontal cortical region by iodixanol density gradient as described above (12 weeks old, 3 mice for each genotype were used). Collected nuclei were incubated with anti-NeuN conjugated with Alexa Fluor 488 (1:1000, Millipore #MAB377X). NeuN(+)-nuclei and NeuN(-)-nuclei were sorted by FACSAria II (BD). After collection of NeuN(+) nuclei, ChIP-seq analysis was conducted as previously reported (Fukuda et al., 2018). In brief, chromatin was immunoprecipitated from isolated NeuN(+) nuclei using H3K9me2 antibody (6D11) after Micrococcal Nuclease treatment. ChIP-seq libraries were prepared using KAPA Hyper Prep Kit (Kapa Biosystems) and sequenced using HiSeq X (Illumina Inc.). Raw FastQ data were trimmed with Trim Galore (v0.3.7, default parameters) and mapped to the mouse GRCm38 genome assembly using Bowtie (v4.4.6) (Langmead and Salzberg, 2012; Langmead et al., 2009). Read per million (RPM) in each 80-kb window was calculated by original perl script. For correlation analysis between H3K9me2 ChIP-seq data in NeuN+ neuron and HiC-seq data in J1 mESCs (Dixon et al., 2012), we used component score in each 80-kb bin, which was gifted from Dr. Hiratani in Riken (Takahashi et al., 2019). Hi-C data derived from J1 mESCs were used for the analysis (Dixon et al., 2012). Cool format Hi-C fragment data sets (https://github.com/mirnylab/cooler) were downloaded (ftp://cooler.csail.mit.edu/coolers, file name: Dixon2012-J1 mESC-HindIII-allreps-filtered.frag.cool) and this .cool format fragment file was converted to 80-kb bin resolution data sets using the cooler tools and an in-house script. Normalization was performed using the balance command of cooler using default parameters. After this, A/B compartments were calculated using the cworld package (https://github.com/dekkerlab/cworld-dekker; matrix2compartment.pl, option: --ez).

#### Gene ontology analysis

For gene ontology enrichment analysis, DE gene (FDR < 0.01 and logFC > 0.9) was analyzed by GSEA (Mootha et al., 2003; Subramanian et al., 2005).

### Quantification and statistical analysis

Statistics analysis was performed using Prism6, R or Microsoft Excel application. All statistics analysis was summarized in Table S6.

## Supplemental information

Document S1. Figures S1–S6, and Tables S1, S4, and S5

Table S2. Up and down-regulated genes in Ehmt1^Δ/+^ mice brain identified by single-nuclear RNA-seq analysis, related to figure 4

Table S3. Gene ontology analysis of up and down-regulated genes in Ehmt1^Δ/+^ mice brain, related to figure 4

Table S6. Statistical analysis results for this study, related to STAR Methods
