# Derepression of inflammation-related genes link to microglia activation and neural maturation defect in a mouse model of Kleefstra syndrome

**DOI:** 10.1016/j.isci.2021.103253

**Published:** 2021-10-19

**Authors:** Ayumi Yamada, Takae Hirasawa, Kayako Nishimura, Chikako Shimura, Naomi Kogo, Kei Fukuda, Madoka Kato, Masaki Yokomori, Tetsutaro Hayashi, Mana Umeda, Mika Yoshimura, Yoichiro Iwakura, Itoshi Nikaido, Shigeyoshi Itohara, Yoichi Shinkai

## Main text

(iScience *24*, 102741-1–102741-20; July 23, 2021)

During the preparation of the revised manuscript, Figure S3 was accidentally duplicated as Figure S2, and Figure S2 was missing from the supplemental information. This has now been corrected online. The authors apologize for any confusion caused to the readers.

## Supplemental information

Doument S1. Figures S1–S7 and Tables S1, S4, and S5

