# Supplementary material for: Derepression of inflammation-related genes link to microglia activation and neural maturation defect in a mouse model of Kleefstra syndrome
Source: iScience. 2021 Jun 17;24(7):102741. doi: 10.1016/j.isci.2021.102741 (PMC8258976; doi:10.1016/j.isci.2021.102741)
Supplement: Document S1. Figures S1–S6, and Tables S1, S4, and S5 [file mmc1.pdf]

iScience, Volume *24*

Supplemental information

Derepression of inflammation-related genes link to microglia activation and neural maturation defect in a mouse model of Kleefstra syndrome

Ayumi Yamada, Takae Hirasawa, Kayako Nishimura, Chikako Shimura, Naomi Kogo, Kei Fukuda, Madoka Kato, Masaki Yokomori, Tetsutaro Hayashi, Mana Umeda, Mika Yoshimura, Yoichiro Iwakura, Itoshi Nikaido, Shigeyoshi Itohara, and Yoichi Shinkai

Supplemental Information

Document S1. Figure S1-S7 and Table S1, S4 and S5

Fig. S1 FLAG-GLP expression in both neuronal and non-neuronal cells of *Ehmt1 iTg* (+ or -) in *Nestin-Cre* mice brain (related Fig. 1)

(A) Genomic structure of inducible *Ehmt1* Tg mice. In the case of *CreER* mouse, oral administration of Tamoxifen (Tam) induced exogenous *FLAG-Ehmt1* expression. (B) FLAG-GLP expression in *Ehmt1* iTg (+); *Nestin-Cre* or iTg (-) mice. Green staining shows FLAG-GLP expression and magenta staining represents each cell type. NeuN for neuron, Olig2 for oligodendrocyte and GFAP for astrocyte were used for cell type specific markers. White arrowhead indicated oligodendrocyte or astrocyte, respectively. DNA was stained with DAPI (cyan). Magnification; X40 with X2 digital zoom

Fig. S2 FLAG-GLP expression in *Ehmt1* iTg; *CAG-CreER* mice (related Fig. 1) or in *Ehmt1* iTg; *CamKII-CreER* mice after Tam treatment (related Fig. 2)

(A) FLAG-GLP expression was induced by Tam administration at 3 and 4 weeks old. FLAG-GLP (Green) was detected in both neuronal and non-neuronal cells of *Ehmt1* iTg; *CAG-CreER* at 5 weeks old mouse cortical region (1 week after induction). Each cell type was detected with cell type specific markers same as Fig. S1B (magenta). White arrowhead indicated oligodendrocyte or astrocyte, respectively. DNA was stained with DAPI (cyan). Magnification; X40. (B) H3K9me2 level of NeuN+ or GFAP+ cells were quantitated and normalized with pan H3 staining. Forty NeuN+ or GFAP+ cells were quantitated and shown as mean±SD (n=3). *$p<0.05$ (vs WT), One-way ANOVA. (C)

FLAG-GLP expression in cortex and hippocampus area after Tam treatment in *Ehmt1* iTg; *CamKII-CreER* mice. FLAG-GLP (green) was only expressed in NeuN+ cells (magenta), FLAG signal was absent in NeuN negative cells (white arrow head). Magnification; X60 with digital zoom X1.5) (D) GLP level (green) of NeuN+ (cyan) or GFAP+ (cyan) cells in cortex area (wild type (WT, circle), *Ehmt1*$^{\Delta/+}$ (Δ/+, square), *Ehmt1* iTg (iTg, up triangle) and *Ehmt1*$^{\Delta/+}$; iTg (Δ/+; iTg, diamond) with *CamKII-CreER* mice) was quantitated and normalized with pan H3 staining (magenta). Thirty cells were quantitated for each animal and relative amount of GLP protein was calculated from mean of WT as 1 and shown as mean±SD (n=3). *p<0.05, **p<0.01 and ***p<0.001 (vs WT), One-way ANOVA. Magnification; X60.

Fig. S3 Level of H3K9me2 in *Ehmt1* iTg; *CamKII-CreER* mouse brain with Tam. (related to Fig. 2)

(A) H3K9me2 (green) and pan-H3 (magenta) staining of wild type (WT), *Ehmt1*$^{\Delta/+}$ (Δ/+), *Ehmt1* iTg (iTg) and *Ehmt1*$^{\Delta/+}$; iTg (Δ/+; iTg) with *CamKII-CreER* mice brain, quantitated graph was shown in Fig 2B. Neurons were stained with anti-NeuN Ab (left, cyan) and astrocytes were stained with anti-GFAP Ab (right, cyan), respectively. Magnification; X60. (B) Comparison of two independent H3K9me2 ChIP experiments (ChIP#1 and #2). ChIP analysis was performed with sorted NeuN+ nuclear fraction prepared from WT, Δ/+, iTg and Δ/+; iTg mice cortex (Tam treatment at 3 weeks old of age, and dissection at 12 weeks old). Pearson's r value was shown at the right bottom corner. (C) H3K9me2 ChIP seq data of experiment #2 was shown as Read per million (RPM) in 80 kb window (upper panel) and compartment score (lower panel) vs WT.

Fig. S4 H3K9me2 ChIP-seq analysis of published adult hippocampus of *Ehmt1+/–* mice (related to Fig. 2) and illustration for microglia classification. (related to Fig. 3C)

(A, B) H3K9me2 ChIP-seq data of adult hippocampus of *Ehmt1*$^{\Delta/+}$ mice reported by (Iacono et al 2018) was re-analyzed and shown as read per million (RPM) in 80 kb window (**A**) and compartment score vs WT (**B**). (C) Illustration and summary table for morphological classification of microglia. Iba-1+ microglia were classified into 5 morphological groups (class I to V) with same discrimination reported by Diz-Chaves et al. (2012).

Fig. S5 Recovery of the *Ehmt1*$^{\Delta/+}$ mouse brain phenotypes by neuron specific GLP supply from the postnatal stage. (related to Fig. 4)

(A) Representative images of Golgi staining of cortex area in *CamKII-CreER* group mice (Tam+, 16 weeks old). Spine morphology surrounded with red-dotted square was enlarged and shown at upper right box. (B) Single nuclear RNA sequencing of nuclei isolated from mouse frontal cortical region using 3 WT, *Ehmt1*$^{\Delta/+}$, *Ehmt1* iTg and *Ehmt1*$^{\Delta/+}$; iTg mice each (14 weeks old). UMAP plots showed the cluster of neuron (green), microglia (pink) and oligodendrocyte (blue), respectively. (C) Histone H3K9me2 ChIP analysis in *Casp1* locus. NeuN+ cells were sorted from wild type (WT, gray), *Ehmt1*$^{\Delta/+}$ (Δ/+, light gray), *Ehmt1* iTg (iTg, dark gray) and *Ehmt1*$^{\Delta/+}$; iTg (Δ/+; iTg, angled stripe) with *CamKII-CreER* mice cortex (12 weeks old). ChIP efficiency of two independent experiments was shown as %input. (D) Microglial cells in cortex area of

CamKII-CreER group mice (Tam+, 16 weeks old) were stained with Iba-1 antibody (magenta) and classified by morphology into type I-V. Magnification; X60

Fig. S6 Summary of GLP supply experiments by different Cre or CreER driver mouse lines (related to Fig. 1-5)

(OF; open field test, LD; light and dark test. IHC; immunohistochemistry, WB; western blot analysis)

Fig. S7 Quality control for single-nucleus RNA seq (related to Fig. 4 and 5D)

The coverage profiles against gene bodies before or after trimming were shown. Poor performing sample were eliminated from analysis. (A) Wild type (WT) and *Ehmt1*$^{\Delta/+}$ mice sample (related Fig. 4) (B) CamKII-CreER series samples (WT, *Ehmt1*$^{\Delta/+}$, *Ehmt1* iTg and *Ehmt1*$^{\Delta/+}$; iTg with *CamKII-CreER* mice treated with Tam at 3 weeks old)

Table S1. Read number of H3K9me2 ChIP-seq analysis (related Fig. 2C and S3B)

Table S4. List of oligonucleotides used in this study (related to STAR Methods)

Table S5. List of genes used to classify cell types for single nucleus RNA-seq analysis (related to STAR Methods)

FigS1_Yamada, A

(A)

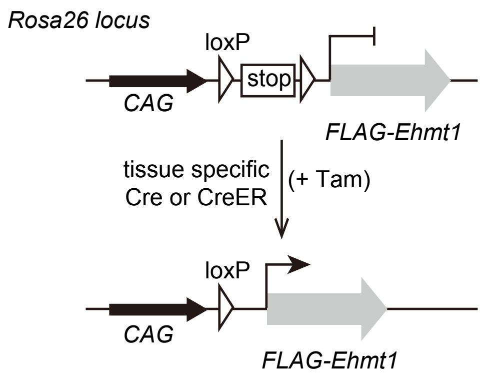

(B)

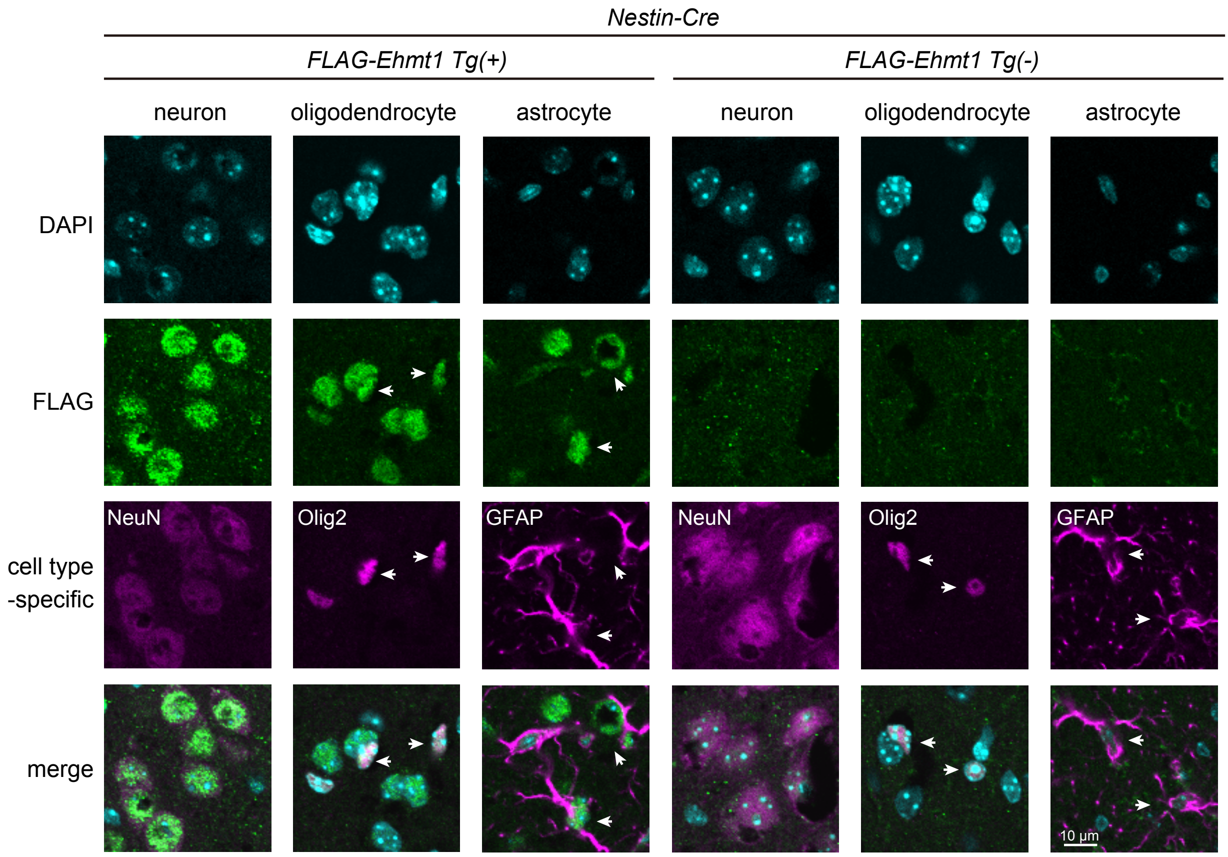

FigS3_Yamada, A

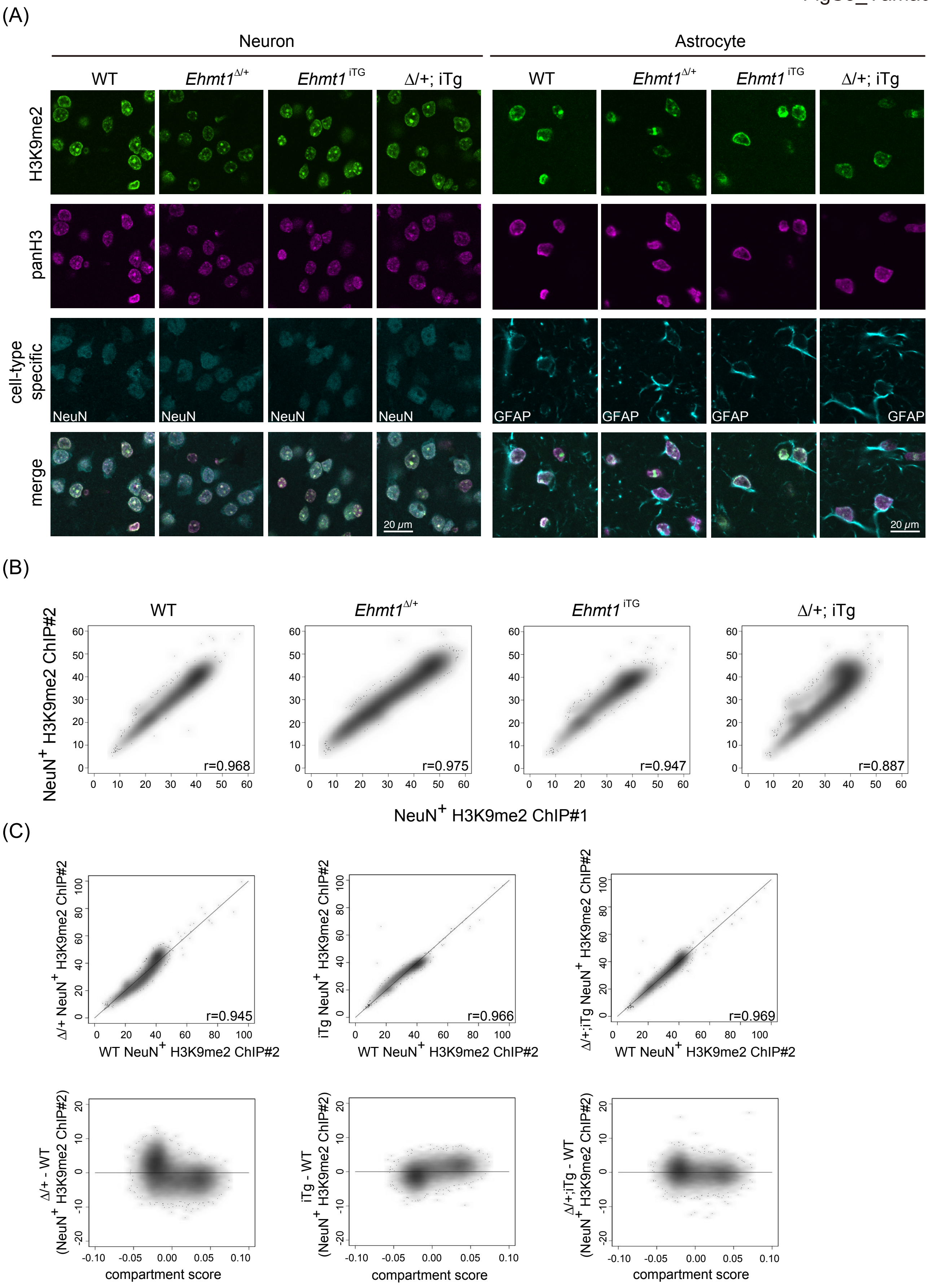

FigS3_Yamada, A

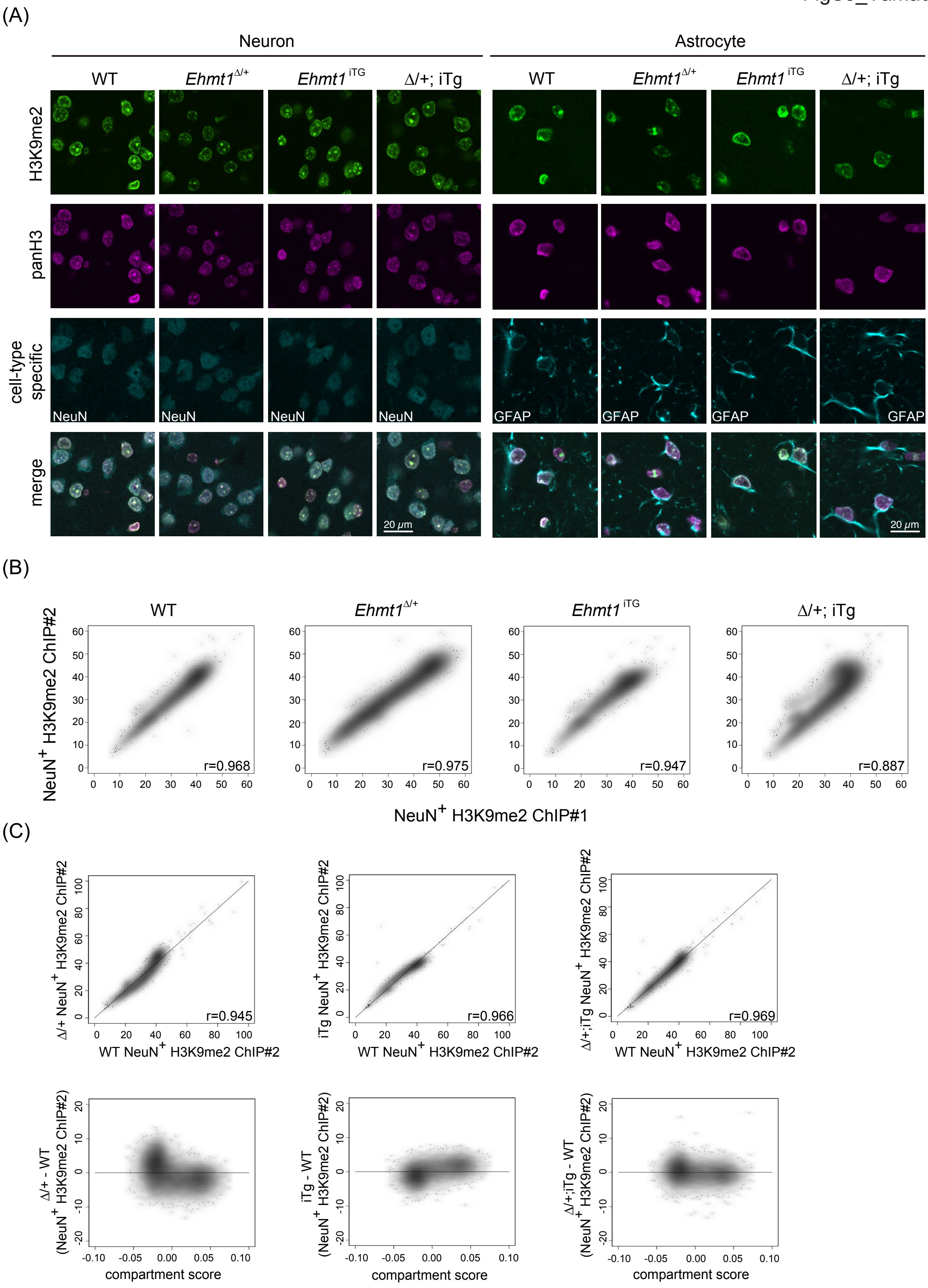

FigS4_Yamada, A

(A)

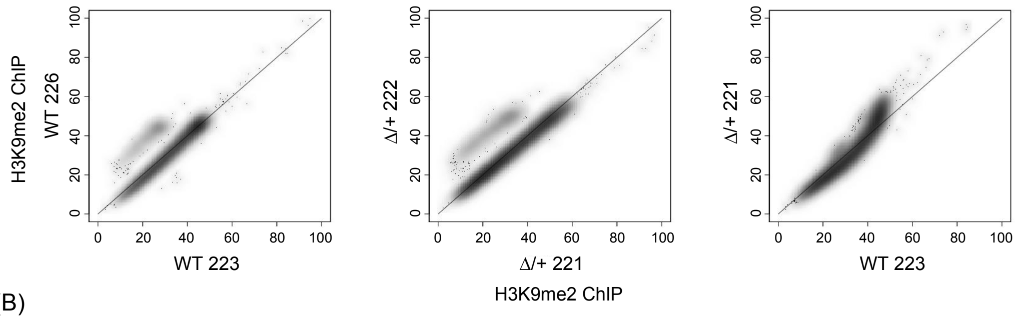

(B)

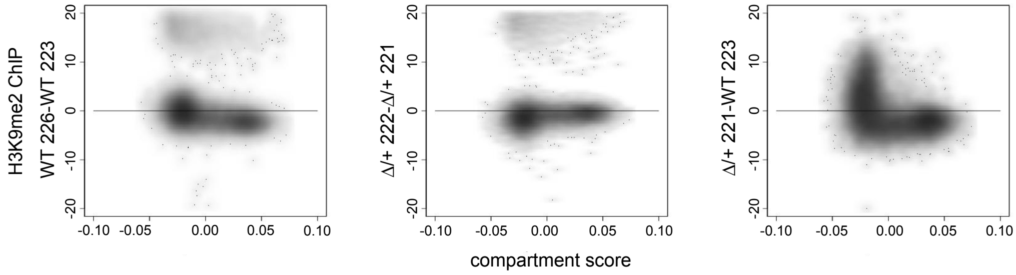

(C)

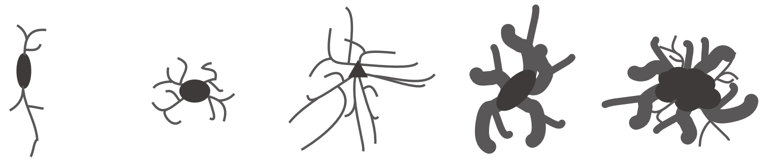

	I	II	III	IV	V
branch number	1-2	<4	many	many	many
branch morphology	thin	thin/short	thin/long	thick/short	thick/short
cell body			small	big	amoeboid

FigS5_Yamada, A

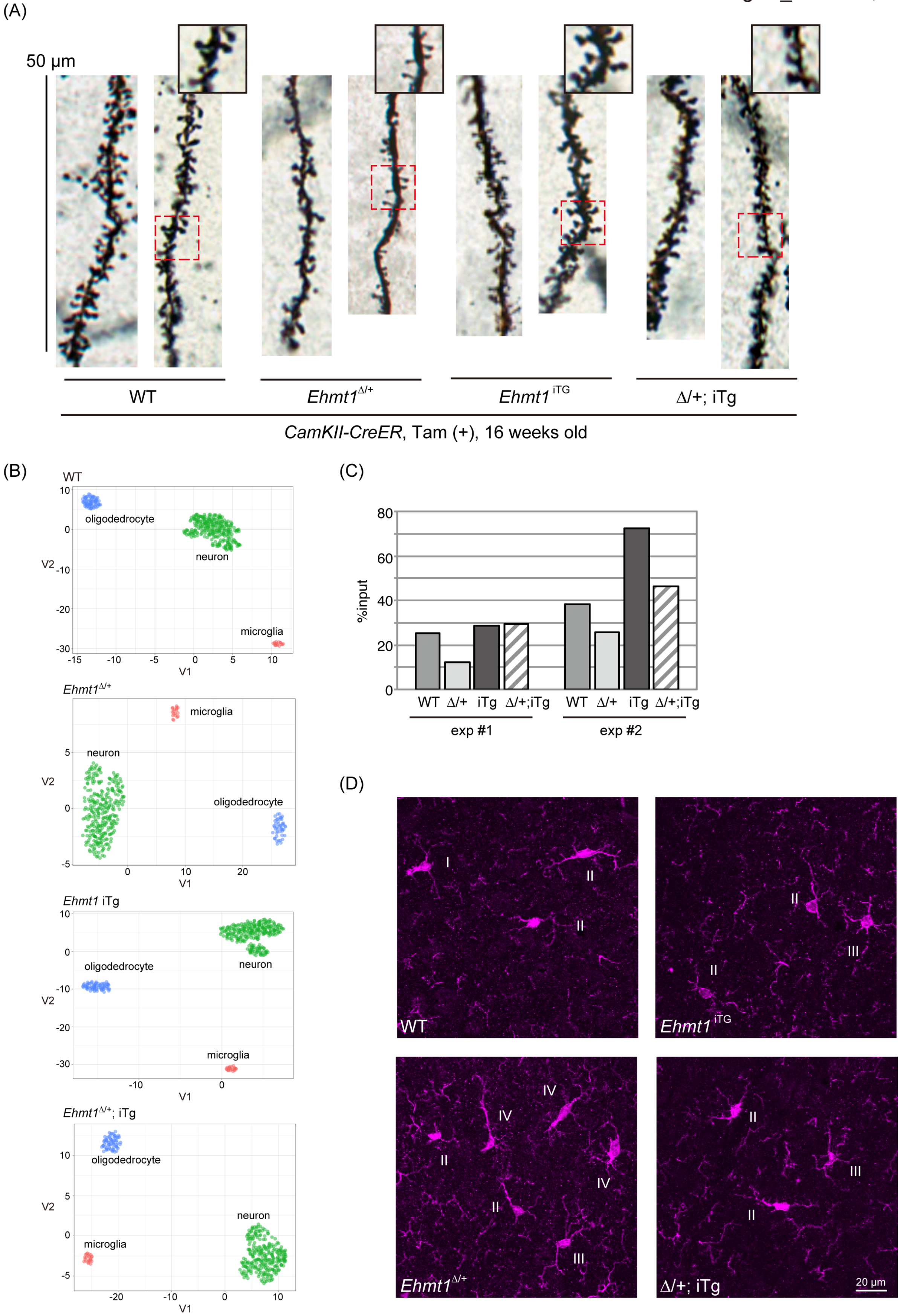

FigS6_Yamada, A

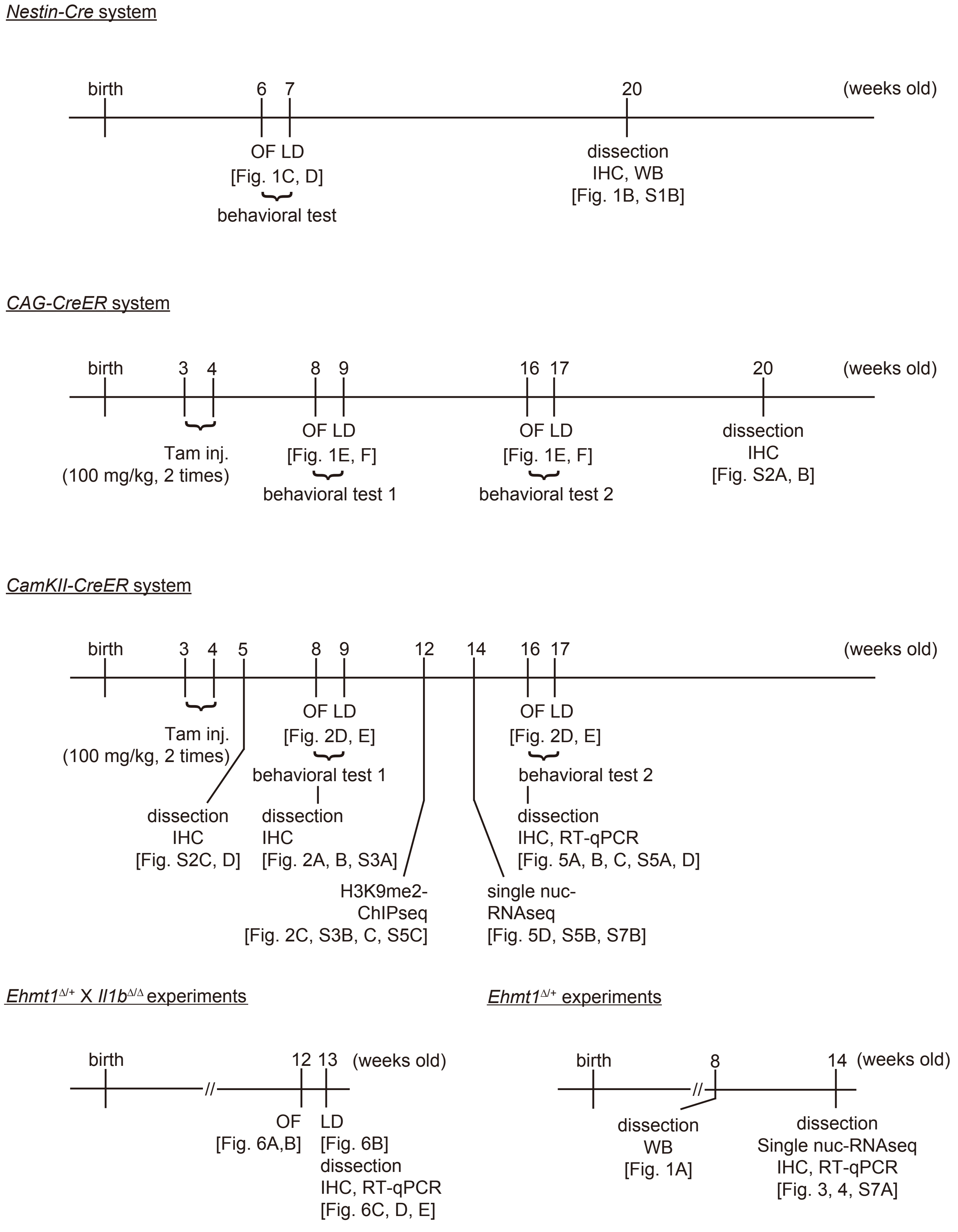

FigS7_Yamada, A

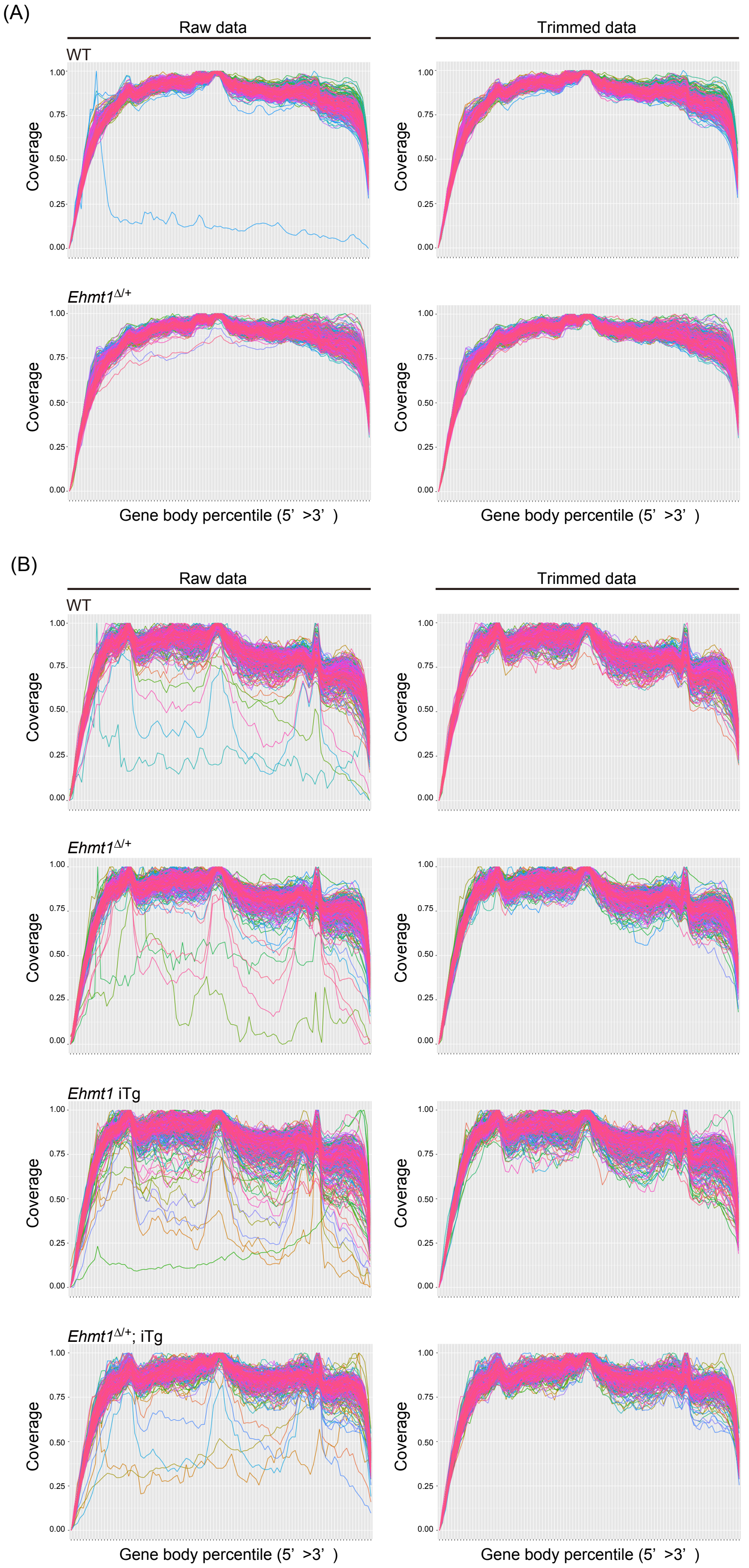

Table S1. Read number of HistoneH3K9-dimethylation ChIP analysis, related Fig. 2C and S3B.

ChIP	genotype	read number		
		total	after remove low quality read	after mapping and remove duplication
#1	WT	69526988	68923154	62708707
	Ehmt1$^{\Delta/+}$	64064010	63448000	41658398
	Ehmt1 iTg	70175498	69385090	47323382
	Ehmt1$^{\Delta/+}$; iTg	78921630	78436226	55552788
#2	WT	69526988	68923154	51564050
	Ehmt1$^{\Delta/+}$	54283362	53869616	40204190
	Ehmt1 iTg	87350172	86649776	53036087
	Ehmt1$^{\Delta/+}$; iTg	65198348	64644578	45069878

Table S4. List of Oligonucleotides used in this study, Related to STAR Methods

Primers used for RT-qPCR

Gene	Forward primer (5' to 3')	Reverse primer (5' to 3')
mCasp1	TGGTCTTGTGACTTGGAGGAC	CCCTATCAGCAGTGGGCATC
mIl1b	ACAAGGAGAACCAAGCAACG	GTGCCGTCTTTCATTACACAGG
mTubb3	TGGACAGTGTTCGGTCTGG	CCTCCGTATAGTGCCCTTTGG
mHprt	CGTGATTAGCGATGATGAACCAGG	CATCTCGAGCAAGTCTTTCAGTCC

Primers used for ChIP-qPCR

Gene	Forward primer (5' to 3')	Reverse primer (5' to 3')
mCasp1	CTTGTTCCTCTCCTGTGAATCC	AAGGAAGGGAGTGAGTCTTAGT

Table S5. List of genes used to classify cell types for single nucleus RNA-seq analysis (related to STAR Methods)

cell type			*Snap25*	*Slc17a7*	*Gad1*	*Rorb*	*Foxp2*	*Ngb*	*Enpp2*	*Ctgf*	*Hsd11b1*	*Bcl6*	*Sox9*	*Olig1*	*Ctss*
neuron	excitatory	L2	+	+				+	+						
		L3	+	+					+						
		L4	+	+		+									
		L5a	+	+		+					+				
		L5b	+	+								+			
		L6a	+	+			+								
		L6b	+	+			(+)			+					
	inhibitory		+		+										
non-neural cell	astrocyte												+		
	oligodendrocyte													+	
	microglia														+
